# Viral sequence analysis of chronic hepatitis B patients treated with the siRNA JNJ-73763989 in phase II clinical trials

**DOI:** 10.1016/j.jhepr.2025.101618

**Published:** 2025-10-09

**Authors:** Thierry Verbinnen, Erkki Lathouwers, John Jezorwski, Michael Biermer, Ilse Augustyns, Craig Grant, Kosh Agarwal, Man-Fung Yuen, Sandra De Meyer, Oliver Lenz

**Affiliations:** 1 Johnson & Johnson, Beerse, Belgium; 2 Johnson & Johnson, Titusville, United States; 3 Johnson & Johnson, High Wycombe, United Kingdom; 4 Institute of Liver Studies, King’s College Hospital, London, United Kingdom; 5 Department of Medicine, The University of Hong Kong, Hong Kong, China

**Keywords:** hepatitis B, siRNA, genome sequencing, JNJ-73763989, DAP/TOM, polymorphisms, viral breakthrough, viral relapse, HBsAg

## Abstract

**Background & Aims:**

JNJ-73763989 (JNJ-3989) is a small-interfering RNA composed of two triggers targeting the HBsAg and HBx protein open reading frame, designed to target all HBV RNAs for degradation. JNJ-3989 + nucleos(t)ide analogue (NA) treatment dose-dependently reduced chronic hepatitis B (CHB) viral antigens. Viral sequence changes in the JNJ-3989 S-/X-trigger target regions were evaluated at baseline, on-treatment, and in patients with virologic relapse (VR) after discontinuation of all treatment in REEF-1 (NCT03982186) and REEF-2 (NCT0412954) studies.

**Methods:**

HBV DNA/RNA was extracted from plasma samples, and the HBV genome was sequenced using next-generation sequencing. Nucleotide variants were defined as changes *vs.* the universal HBV reference sequence (read frequency >15%).

**Results:**

Baseline polymorphisms in the JNJ-3989 target region complementary to positions 2-18 of the S-/X-trigger were present in 10.1% and 2.4% of not currently treated patients, respectively, with no relevant impact on JNJ-3989-induced HBsAg decline. Variants at X-trigger target region positions of interest (POI) were more frequently observed off treatment in JNJ-3989-treated virologically suppressed (VS) patients with VR *vs*. NA-control arm VS patients with VR (55.8% *vs*. 5.7%, respectively). Variants at S-trigger POI were observed off treatment in 32.1% and 19.4% of JNJ-3989- and NA-control treated VS patients with VR, respectively. Off-treatment HBsAg kinetics did not differ between JNJ-3989-treated patients with and without variants in S-/X-trigger target POI during VR.

**Conclusion:**

Baseline sequence polymorphisms did not impact JNJ-3989 treatment response. JNJ-3989-treated patients who experienced VR post-treatment had variants in the X-trigger target region during VR, but not at baseline or on-treatment, suggesting that X-trigger variants developed off treatment in JNJ-3989-treated patients. The presence of these variants did not impact off-treatment HBsAg kinetics.

**ClinicalTrial.gov Identifiers:**

NCT03982186 and NCT0412954.

**Impact and implications:**

Small-interfering RNA (siRNA) therapy with JNJ-3989 in patients with chronic hepatitis B induces potent, dose-dependent reductions in viral antigens, though the magnitude of decline varies among individuals. Baseline nucleotide polymorphisms in JNJ-3989 trigger target regions did not account for variability in HBsAg or HBeAg responses. Substitutions in siRNA trigger target regions were frequently detected during and after virologic relapse in JNJ-3989-treated patients, confirming antiviral target engagement. However, the emergence of these variants did not affect off-treatment HBsAg or HBV DNA kinetics. This study provides the first comprehensive clinical virology analysis of siRNA-based therapy in HBV infection, offering insights relevant to the broader development of antiviral siRNA therapeutics. The clinical significance of X-trigger substitutions for potential re-treatment with JNJ-3989 or other HBV-targeting siRNAs remains to be determined.

ClinicalTrial.gov Identifiers: NCT03982186 and NCT0412954.

## Highlights

• JNJ-3989 S- and X-trigger target region baseline polymorphisms were present in 10.1% and 2.4% of patients with CHB.
• No impact of these baseline polymorphisms on JNJ-3989 induced HBsAg and HBeAg declines.
• JNJ-3989-treated patients with viral relapse frequently had substitutions within the X-trigger target region.
• These X-trigger substitutions likely emerged following increased HBV replication after stopping all treatment.
• The detection of S-and X-trigger substitutions was not associated with differences in off-treatment HBsAg kinetics.

## Graphical abstract

**Figure:**
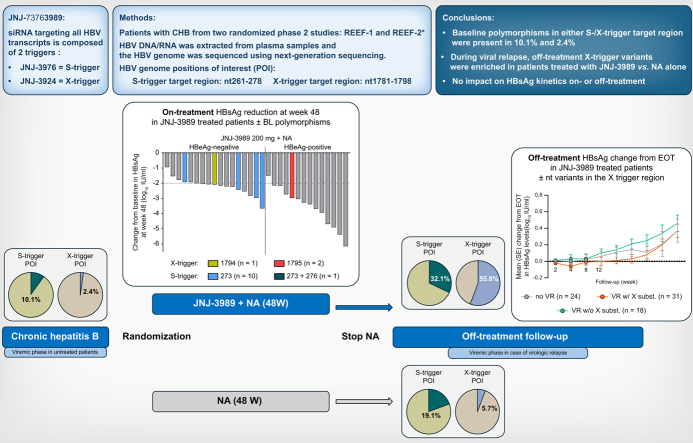

## Introduction

JNJ-73763989 (JNJ-3989) is a liver-targeted small-interfering RNA (siRNA) designed to target all HBV RNAs for degradation, thereby reducing all HBV viral proteins and pregenomic RNA.^1^ JNJ-3989 is composed of two triggers, JNJ-3976 (“S-trigger”) and JNJ-3924 (“X-trigger”), both synthetic, 21-nucleotide (nt) long, double-stranded siRNA molecules targeting the hepatitis B surface antigen (HBsAg) and hepatitis B x protein open reading frames, respectively. The phase IIb REEF-1 (NCT03982186) and REEF-2 (NCT0412954) studies showed that JNJ-3989-based treatment was well tolerated by patients with chronic hepatitis B (CHB) and led to JNJ-3989 dose-dependent reductions in all viral markers.^2^^,^^3^

Sequence-specific siRNAs, like JNJ-3989, contain a 5' (nucleotide position 2-8) seed region critical for target mRNA recognition, and a 3' supplemental region for binding stability and specificity. Effective silencing requires a (near-)perfect match between seed region and RNA target.[4], [5], [6], [7] Pre-existing and emerging viral resistance to direct antiviral drugs, resulting in lack of activity and subsequent treatment failure, is well documented.[8], [9], [10] Our understanding of viral resistance to viral genome targeting siRNAs is mainly restricted to *in vitro* data, given limited experience with this type of agent in the clinic.[11], [12], [13]

Here we assessed viral sequence changes within the S-/X-trigger target regions in HBV-infected patients enrolled in REEF-1 and REEF-2, focusing on viral sequence variants present either prior to the start of JNJ-3989 treatment or observed in post-baseline samples from patients with virologic relapse. In addition, the impact of these viral sequence substitutions on response to JNJ-3989 treatment was evaluated.

## Patients and methods

### Study design and patients

REEF-1^3^ (Fig S1) and REEF-2^2^ (Fig S2) were randomized, double-blind, multi-center, placebo-controlled studies of patients with CHB who received 48 weeks of treatment with JNJ-3989/placebo + NA (daily; oral) followed by 48 weeks of follow-up. Patients in REEF-1 were eligible to stop NA treatment if they met pre-defined NA stopping criteria (HBsAg <10 IU/ml, HBV DNA <LLOQ (lower limit of quantification), hepatitis B e antigen (HBeAg)-negative, alanine aminotransferase [ALT] <3 × the upper limit of normal) at Week 48 or during 48-weeks follow-up. Patients in REEF-2, all virologically suppressed (VS) at baseline, stopped NA treatment at Week 48. Patients stopping all treatment were monitored for pre-defined NA re-treatment criteria based on increases in HBV DNA and/or ALT.

Both studies were conducted in full compliance with the Declaration of Helsinki and Good Clinical Practice guidelines. All patients provided written informed consent.

### Viral sequence analysis

#### HBV DNA-based full genome sequencing

HBV DNA was extracted from plasma samples with sufficiently high viral DNA levels, and the full HBV DNA genome was sequenced using Illumina MiSeq sequencing (Illumina, San Diego, CA, USA) with a 1% sequence read cut-off (supplementary methods).^14^

Viral sequence analyses focused on nt changes in the HBV target regions complementary to positions 2-18 of the JNJ-3989 S- (nt261-278) and X-trigger (nt1781-1798), which are considered potentially relevant for siRNA target binding and activity.^15^^,^^16^

#### Exploratory HBV RNA-based sequencing

Baseline samples from REEF-1 VS patients and a subset of Week 48 samples from REEF-1 not currently treated (NCT) patients were sequenced using plasma HBV RNA. The HBV RNA reverse-transcription PCR used for sequencing was based on a previously described assay aimed to detect the polyadenylated 3’end of all covalently closed circular DNA-derived HBV RNA transcripts, including pregenomic RNA (supplementary methods).^17^

## Results

### Baseline polymorphisms and impact on serologic response

Baseline polymorphisms in the target region complementary to positions 2-18 of the JNJ-3989 S-/X-triggers were present in 17/168 (10.1%) and 4/165 (2.4%) NCT REEF-1 patients, respectively (Table S1). Most patients had single nt polymorphisms at S-trigger position of interest (POI) 273, and two each had a single nt polymorphism at X-trigger POI 1794 or 1795. One patient had a combination of nt polymorphisms at S-trigger POI 273 and 276.

Due to low or negative HBV DNA levels in VS patients, baseline samples of REEF-1 VS patients were sequenced using plasma HBV RNA as starting material. Five of 36 (13.9%) and 0/41 (0.0%) REEF-1 VS patients with baseline HBV RNA-based sequence data available had polymorphisms at S-/X-trigger POI, respectively; four had a single nt polymorphism (A273G) and one had A273G+T276 C/T polymorphisms (Table S1).

**Fig. 1:**
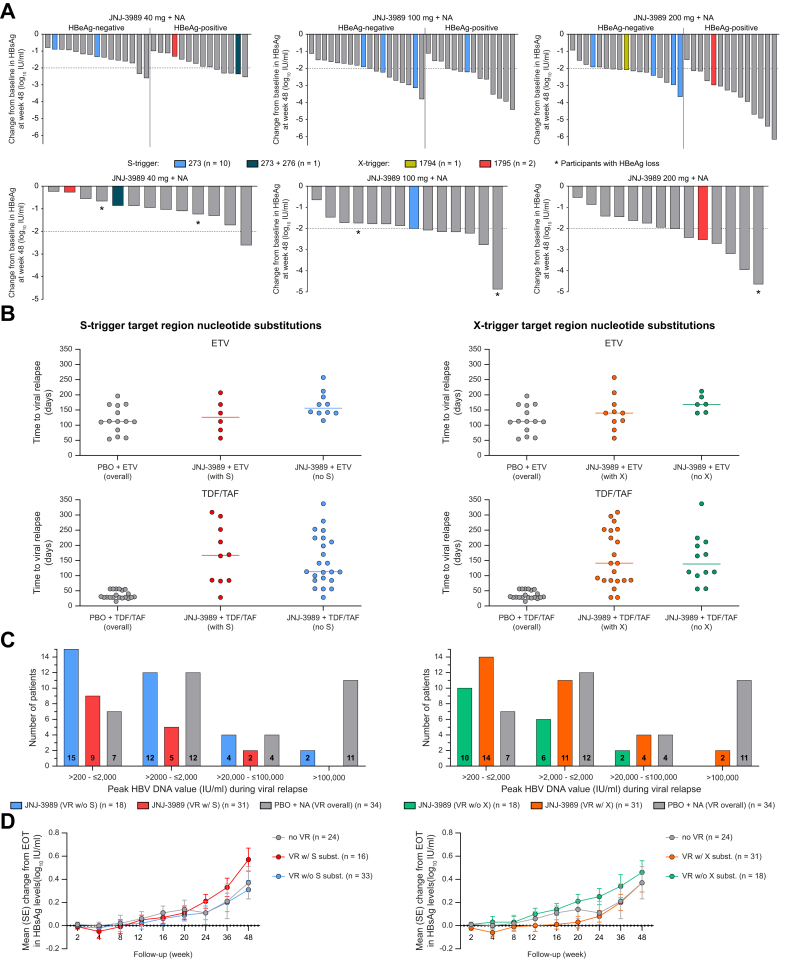
HBV antigen responses and HBV kinetics by presence of S- and X-trigger target region nucleotide substitutions. (A) Only NCT participants who completed 48 weeks of JNJ-3989 + NA treatment and had S- and X-gene sequence data available at baseline. (B) Patients with VR (*i.e*., confirmed off-treatment HBV DNA >200 IU/ml) and off-treatment sequence data available. (C) Peak HBV DNA category (*i.e*., >200, >2,000, >20,000, and >100,000 IU/ml) had to be confirmed at ≥2 consecutive off-treatment time points or measured at the last single off-treatment time point. (D) Mean (SE) change from EOT in HBsAg levels by off-treatment VR and presence or absence of either S- or X-trigger target region substitutions. BL, baseline; EOT, end of treatment; ETV, entecavir; HBeAg, hepatitis B e antigen; HBsAg, hepatitis B surface antigen; HBV DNA, hepatitis B DNA; NA, nucleos(t)ide analogue; NCT, not currently treated; ND, not determinable; PBO, placebo; POI, positions of interest (nt 261-278 for S-trigger and nt1781-1798 for X-trigger); SCR, Screening; SE, standard error; Subst, nt-substitution; TDF/TAF, tenofovir; TW, treatment week; VR, viral relapse; VS, virologically suppressed; w/, with; w/o, without.

Comparison of the individual HBe/sAg declines from baseline for patients with and without baseline polymorphisms confirmed that baseline sequence polymorphisms in the siRNA target regions did not impact serologic response to JNJ-3989-based treatment (Fig. 1A).

### Viral sequencing analysis of patients with VR

Post-baseline HBV DNA-based S-/X-gene sequence data were available from >90% of VS patients with VR (Table 1). Among VS patients with VR, X-trigger target region nt substitutions were more frequently observed during off-treatment follow-up visits in JNJ-3989 ± JNJ-6379-treated patients than in NA-treated patients (55.8% and 5.7%, respectively; Table 1). More specifically, nt substitutions were observed at X-trigger target POI 1784, 1785, 1794, and 1795 at a range of 5.2-42.9% among JNJ-3989 ± JNJ-6379-treated VS patients with VR and at a range of 0.0-2.9% among NA-only-treated VS patients with VR (Tables 1 and S2). S-trigger target region nt substitutions were observed during off-treatment follow-up visits in 32.1% of JNJ-3989-treated VS patients and 19.4% of NA-only-treated VS patients with VR (Tables 1 and S3). Most had substitutions at S-trigger POI 273.

**Table 1:** Proportion of VS patients with VR and nucleotide substitutions observed at ≥1 S- and X-trigger target region POI at time of VR and during aggregated off-treatment follow-up period – by study and treatment.

	Total REEF-1 & REEF-2 JNJ-3989 (±JNJ-6379) + NA		REEF-2 NA-control arm	
VS patients, mITT, n	321		44	
Patients who stopped all treatment (including NA), n	127		40	
Patients with VR, n (%)	75 (59.1)		34 (85)	
Patients with HBV DNA >200 IU/ml at single off-treatment time point, n (%)	9 (7.1)		5 (12.5)	

**Time point of viral sequencing**	**VR + 12w**†	**Aggregated FU^‡^**	**VR + 12w**†	**Aggregated FU^‡^**
Patients with S-gene sequence info available, n	70	78	33	36
≥1 substitutions at S-trigger POI 2-18, n (%)∗	17 (24.3)	25 (32.1)	5 (15.2)	7 (19.4)
264	1 (1.4)	1 (1.3)	-	-
273	16 (22.9)	22 (28.2)	5 (15.2)	7 (19.4)
276	1 (1.4)	3 (3.8)	-	1 (2.8)
Patients with X-gene sequence info available, n	69	77	33	35
≥1 substitutions at X-trigger POI 2-18, n (%)∗	21 (30.4)	43 (55.8)	2 (6.0)	2 (5.7)
1784	2 (2.9)	5 (6.5)	-	-
1785	4 (5.8)	14 (18.2)	-	-
1787	-	1 (1.3)	-	-
1793	1 (1.4)	1 (1.3)	-	-
1794	15 (21.7)	33 (42.9)	1 (3.0)	1 (2.9)
1795	-	4 (5.2)	-	-
1796	-	1 (1.3)	-	-
1797	-	-	1 (3.0)	1 (2.9)

EOT, end of treatment; FU, follow-up; mITT, modified intent-to-treat; NA, nucleos(t)ide analogue; nt, nucleotide; POI, position of interest; VR, viral relapse (*i.e*., confirmed off-treatment increase in HBV DNA >200 IU/ml in patients with HBV DNA <LLOQ at EOT); VS, virologically suppressed.

Observed variants are defined as changes from the universal HBV genotype A (NCBI ID X02763) reference sequence with sequence read frequency was >15%.

Viral sequencing data included in the analysis was considering either i) the first assessment up to and including 12 weeks after viral flare was identified (^†^VR+12w), or ii) all off-treatment time points with sequencing information available (^‡^Aggregated FU).

∗ A single patient could have one or more nucleotide substitutions observed at one or more S- or X-trigger POI during the aggregated off-treatment follow-up period.

† In REEF-2, 7/84 active arm and 4/44 control arm patients either did not stop NA or discontinued study treatment early.^6^

### Exploratory HBV RNA-based sequencing of on-treatment week 48 samples

To evaluate if S-/X-trigger target region variants were already emerging during JNJ-3989 treatment, Week 48 samples from JNJ-3989-treated REEF-1 NCT patients were analyzed. Because most NCT patients achieve HBV DNA <LLOQ early on-treatment,^3^ HBV RNA-based sequencing was performed on Week 48 plasma samples.

Week 48 S- and X-gene regions were successfully sequenced for 25/30 (83.3%) and 28/30 (93.3%) patients, respectively, who had a Week 48 HBV RNA level of ∼3.0 log_10_ copies/ml or higher (data on file). None of these patients with paired baseline (HBV DNA-based) and Week 48 (HBV RNA-based) sequence data had emerging substitutions at any of the S-/X-trigger POI.

### Off-treatment response in REEF-2 patients with S- and/or X-trigger target region variants observed during VR

There were no differences in median time to VR between JNJ-3989-treated patients with or without S-/X-trigger target region substitutions (Fig. 1B, Table S4).

Among JNJ-3989-treated patients, S-/X-trigger target region variants were generally detected at similar frequency in those reaching high or low peak HBV DNA values during VR (Fig. 1C). All three active arm patients with biochemical flare (including one patient with peak HDV DNA >100,000 IU/ml) had X-trigger but no S-trigger target region substitutions during VR (data on file).

There was no relevant difference between off-treatment HBsAg kinetics of JNJ-3989-treated patients with or without VR, and between patients with or without S-/X-trigger target region substitutions observed during or after VR (Fig. 1D).

## Discussion

JNJ-3989-based treatment led to potent and dose-dependent declines in viral antigens in patients with CHB.^2^^,^^3^ However, substantial inter-patient variability in the magnitude of antigen declines was observed. After stopping JNJ-3989 treatment, HBsAg levels increased in most patients but with different kinetics, and with a relevant proportion of patients maintaining sustained suppressed HBsAg levels.^2^^,^^18^ Here, we aimed to determine if pre-existing viral sequence substitutions and post-baseline sequence changes influenced response to JNJ-3989 treatment. Our study represents the first comprehensive clinical virology analysis for siRNA-based therapy in HBV and other viral diseases.

Baseline polymorphisms in the siRNA target regions were infrequent, and HBsAg declines in NCT patients with these variants were comparable to those without variants, suggesting no relevant effect of these sequence variants on JNJ-3989 activity.

Viral sequencing at time of viral relapse showed that most JNJ-3989-treated patients with VR had substitutions at positions complementary to siRNA antisense positions in either the seed (1794 and 1795) or supplemental region (1784 and 1785) of the X-trigger siRNA. As most patients who discontinued NA treatment and were therefore at risk of VR were VS due to long-term NA therapy at study entry, assessment of whether X-trigger substitutions emerged post-baseline could only be made indirectly. Based on indirect comparison of baseline and available Week 48 sequences, as well as public sequence databases (Table S5), it can be concluded that these variants emerged off treatment secondary to HBV DNA increases in JNJ-3989-treated patients who stopped all treatment (see Fig. S5 for representative examples). S-trigger target-region variants were observed at similar frequency in baseline samples of NCT patients, and in samples of JNJ-3989-treated and NA-only treated patients with VR, suggesting S-trigger variants were not newly emerging in patients with VR. The reason for the difference in the emergence of S-/X-trigger target region variants might be due to a lower barrier to resistance of the X-gene region, coding for the HBV X protein only, whereas the S-gene region codes for multiple HBV proteins.^19^ Intrahepatic samples from JNJ-3989-treated patients 28-weeks after last JNJ-3989 injection showed that both S-/X-triggers were still measurable at similar concentrations.^20^^,^^21^ The backbone NAs entecavir and tenofovir, as well as the CAM-E JNJ-6379 are eliminated faster than the siRNA triggers.[22], [23], [24] These subtle differences in the elimination rates between the JNJ-3989-triggers and the small molecule antiviral agents could result in a tail of low-dose JNJ-3989 monotherapy during follow-up, facilitating selection of JNJ-3989 variants. Further evaluation is needed to determine if these X-trigger target region substitutions result in reduced *in vitro* susceptibility to JNJ-3989 treatment. The lack of a suitable *in vitro* HBV system to perform resistance testing is a challenge in the field. A recently published RNA launch system enables the study of HBV replication and could help identify sequence variants that confer resistance to antiviral drugs, including antisense oligonucleotides.^25^

Importantly, the detection of S-/X-trigger substitutions in JNJ-3989-treated patients was not associated with differences in off-treatment HBsAg kinetics. In addition, there was no difference in the time to VR between JNJ-3989-treated patients with and without observed S-/X-trigger variants. Importantly, HBV DNA increases were less frequent and less pronounced in REEF-2 active arm patients *vs.* NA-control arm patients, resulting in a lower rate of ALT flares.^2^ Notably, the detection of S-/X-trigger target variants did not lead to more patients experiencing VR with higher peak HBV DNA values, requiring NA treatment to restart.

In conclusion, baseline nucleotide polymorphisms in the JNJ-3989 S-/X-trigger target regions, relevant for siRNA-induced RNA silencing, were found in 10% and 2.4% of NCT patients, respectively. These polymorphisms did not explain the variability in HBsAg and HBeAg declines during JNJ-3989 treatment. In patients who discontinued treatment and experienced VR, X-trigger substitutions were observed post-VR, likely arising from increased HBV replication after treatment cessation, without affecting off-treatment HBsAg kinetics. JNJ-3989 (DAP/TOM) is under investigation alongside the antisense oligonucleotide bepirovirsen in the B-United study (NCT06537414). The effects of X-trigger substitutions on re-treatment with JNJ-3989 or other RNA-based therapies remain unknown.

## Abbreviations

ALT, alanine aminotransferase; CHB, chronic hepatitis B; HBeAg, hepatitis B e antigen; HBsAg, hepatitis B surface antigen; JNJ-3989, JNJ-73763989; JNJ-6379, JNJ-56136379; NA, nucleos(t)ide analogue; NCT, not currently treated; nt, nucleotide; POI, position of interest; siRNA, small-interfering RNA; VR, viral relapse; VS, virologically suppressed.

## Financial support

This study was sponsored by Johnson & Johnson with medical writing support provided by Lumanity Communications Inc., which was funded by 10.13039/100004331Johnson & Johnson.

## Authors’ contributions

All authors were involved in the critical revisions of the manuscript and the review of important content, were accountable for all aspects of the work (accuracy and integrity), and approved the final submitted manuscript. At least one author had access to all of the data and can vouch for the integrity of the data analyses. TV, EL, JJ, MB, IA, CG, SDM, and OL designed the protocol and analyzed the data.

## Data availability

The data sharing policy of Janssen Pharmaceutical Companies of Johnson & Johnson is available at https://www.janssen.com/clinical-trials/transparency. As noted on this site, requests for access to the study data can be submitted through Yale Open Data Access (YODA) Project site at http://yoda.yale.edu.

## Conflicts of interest

TV, EL, JJ, MB, IA, CG, SDM, and OL are or were employees of Johnson & Johnson and may hold stock in Johnson & Johnson. OL is current employee of GSK and may hold stock in GSK. KA received grants from Abbott and MSD; served as a consultant for Johnson & Johnson, Assembly, Arbutus, Immunocore, Roche, BMS, Boehringer Ingelheim, Novartis, Shinoigi, and Sobi; served as a speaker for Gilead and Sobi; and served on a data safety monitoring or advisory board for Drug Farm, NUC-B, and Aligos. M-FY served as advisor/consultant for AbbVie, AlloVir International, Arbutus Biopharma, Bristol Myers Squibb, ClearB Therapeutics, Dicerna Pharmaceuticals, Gilead Sciences, GlaxoSmithKline, Johnson & Johnson, Merck Sharp & Dohme, Roche, and Spring Bank Pharmaceuticals; and received grant/research support from Assembly Biosciences, Arrowhead Pharmaceuticals, Bristol Myers Squibb, Fujirebio Diagnostics Inc., Gilead Sciences, Merck Sharp & Dohme, Roche, Spring Bank Pharmaceuticals, and Sysmex Corp.

Please refer to the accompanying ICMJE disclosure forms for further details.
