# Supplementary material for: Viral sequence analysis of chronic hepatitis B patients treated with the siRNA JNJ-73763989 in phase II clinical trials
Source: JHEP Rep. 2025 Oct 9;7(12):101618. doi: 10.1016/j.jhepr.2025.101618 (PMC12682118; doi:10.1016/j.jhepr.2025.101618)
Supplement: Multimedia component 1 [file mmc1.pdf]

Viral sequence analysis of chronic hepatitis B patients treated with the siRNA JNJ-73763989 in phase II clinical trials

Thierry Verbinnen, Erkki Lathouwers, John Jezorwski, Michael Biermer, Ilse Augustyns, Craig Grant, Kosh Agarwal, Man-Fung Yuen, Sandra De Meyer, Oliver Lenz

Table of contents

Supplementary methods……………………………………………………………………2

Supplementary tables…………………………………………………………….….…...5

Supplementary figures…………………………………………………………………14

Supplementary references……………………………………………………………..19

Supplementary methods

HBV Genome Sequencing and Genotyping

HBV genome sequencing was performed at DDL Diagnostic Laboratory/Cerba Research (the Netherlands). Total nucleic acids were isolated from 200 µL of plasma and eluted in 20-50 µL of eluate. The isolated DNA was amplified by PCR and nested PCR, using the Expand high-fidelity PCR kit (Roche Molecular Systems). 10 µL of DNA was used for outer PCR. Primers for HBV full genome amplification are in the preCore and the X-gene. If the initial amplification for the full genome large fragment failed, an alternative approach, amplifying the HBV full genome in 2 smaller fragments (HBV full genome partial 1/2) was tested. PCR products were analyzed on a Qiaxel capillary system using standard protocols, to confirm successful amplification of a PCR fragment of the expected size, and to determine whether the quantity of product is sufficient for successful sequence analysis. The PCR products that showed a clear band of the expected size after gel-electrophoresis were cleaned up by Ampure XP beads (Beckman Coulter) to remove primer-dimers and small aspecific PCR products. The purified PCR products were quantified using the Quant-iT PicoGreen dsDNA kit (Life Technologies). The diluted PCR products were fragmented and tagged using the 'tagmentation' method (Nextera XT sample preparation kit). Index primers (Nextera XT Index kit) were added by limited cycle PCR. Prior to sample pooling, samples were normalized using beads with maximum binding capacity (Nextera XT sample preparation kit). The different PCR products were not pooled but sequenced separately. Sequence data from multiple overlapping fragments were subjected to primer trimming and subsequent in-silico pooling before further analysis. Sequencing was performed on the Illumina Miseq platform using the Miseq v2 sequencing kit with 300 cycles (Illumina). De-multiplexed FASTQ files were generated as an output.

Apart from baseline samples, which were sequenced for all patients with sufficiently high HBV DNA levels, sequencing of post-baseline samples was initiated based on changes in

HBV DNA levels observed in each patient and the limits of the sequencing assay. If both the main and the back-up HBV full genome sequence approaches described above failed, exploratory sub-genomic sequencing approaches were attempted. These consisted of either the separate sequencing of partial 1 or partial 2 fragments, the precore/core region, and/or an exploratory short fragment 3 (SF3) (Figure S3).

Baseline polymorphisms included amino acid and nucleotide changes from the universal HBV genotype-A reference sequence (National Center for Biotechnology Information ID X02763) at baseline with a sequence-read frequency ≥15%. Emerging amino acid and nucleotide substitutions included substitutions detected post-baseline with a sequence-read frequency ≥15%, while not present at baseline (read frequency <1%). The sequence-read frequency cutoffs used were consistent with those proposed by the Food and Drug Administration (1).

HBV RNA-based Sequencing and HBV Genotyping

HBV RNA-based sequencing and genotyping was performed at DDL Diagnostic Laboratory/Cerba Research (the Netherlands). Genotyping of HBV-RNA was performed by analysis of the complete RT-domain, which contains sufficient sequence variation to allow identification of all specific HBV GT (Figure S4). After total nucleic acid extraction from the serum or plasma sample, reverse transcription of the RNA was performed by using an HBV-RNA specific RT-primer. Amplification of the RT-domain was performed in two consecutive amplification rounds. Positive RT-domain amplimers were sequenced by ultra-deep sequencing (UDS by Illumina MiSeq), and the HBV GT was identified by phylogenetic analysis comparing the (25% consensus) sequence to HBV GT reference sequences.

The analytical and clinical sensitivity of the assay was tested on dilution series of HBV in vitro transcribed (IVT) RNA, synthesized from HBV plasmid DNA (GT-D), and of 5 plasma samples with known historical HBV GT-A/B/C/D/F. IVT RNA and plasma samples could be amplified

and genotyped when HBV RNA levels were ≥ 2.5 log10 cps/mL. HBV RNA-based GT could be established in 23 of 26 clinical samples (Figure S4) with HBV-RNA concentrations ranging from 2.4 – 6.0 log10 cps/mL (3 failed samples had HBV RNA < 3.5 log10 cps/mL). Majority of samples tested (n=21) had HBV-DNA concentrations < 65 IU/mL, 5 samples had > 20,000 IU/mL.

Comparison of the HBV-RNA-based and prior determined HBV DNA-based GT result showed perfect match. The percentage identity between the HBV-DNA and RNA derived RT-domain sequences ranged between 99.3% and 100%.

Supplementary tables

Table S1: REEF-1 Patients with Baseline Nucleotide Polymorphisms in the JNJ-3989 S- and/or X-Trigger Target Region POIs 2-18 - by Treatment History and HBeAg Status

	NCT / HBeAg-positive	NCT / HBeAg-negative	Total NCT	VS / HBeAg-positive	VS / HBeAg-negative	Total VS
ITT	75	97	172	67	231	298
Patients with S-gene sequencing data, n	75†	93†	168†	26*	10*	36*
No baseline polymorphism, n (%)	71 (94.7)	80 (86.0)	151 (89.9)	26	5 (50.0)	31 (86.1)
1 or more baseline polymorphism, n (%)	4 (5.3)	13 (14.0)	17 (10.1)	0	5 (50.0)	5 (13.9)
273						
A273A/G	3 (4.0)	9 (9.7)	12 (7.1)	-	1 (10.0)	1 (2.8)
A273G	-	4 (4.3)	4 (2.4)	-	3 (30.0)	3 (8.3)
273+276				-		
A273A/G+T276T/C	1 (1.3)	-	1 (0.6)	-	-	-
A273G+T276T/C	-	-	-	-	1 (10.0)	1 (2.8)
Patients with X-gene sequencing data, n	75†	90†	165	28*	13*	41
No baseline polymorphism, n (%)	73 (97.3)	88 (97.8)	161 (97.6)	28 (100)	13 (100)	41 (100)
1 or more baseline polymorphism, n (%)	2 (2.7)	2 (2.2)	4 (2.4)	0 (0.0)	0 (0.0)	0 (0.0)
1794 (T1794T/A)	-	2 (2.2)	2 (1.2)	-	-	-
1795 (T1795T/A)	2 (2.7)	-	2 (1.2)	-	-	-

HBeAg, hepatitis B e antigen. ITT, intent-to-treat. NCT, not currently treated. POI, position of interest. VS, virologically suppressed.

The reference sequence used was the HBV genotype A universal reference sequence (NCBI ID X02763). Baseline polymorphisms were defined as changes from the universal HBV genotype A (NCBI ID X02763) reference sequence and if sequence read frequency was >15%.

The S- and X-trigger target region POI within the S- and X-genes were nt261-278 and nt1781-1798 (HBV full genome numbering based on EcoRI restriction site), respectively.

†HBV DNA-based sequencing was applied to baseline samples of NCT patients (ie, with HBV DNA >2,000 [HBeAg-negative] and >20,000 IU/mL [HBeAg-positive] at screening).

*HBV RNA based sequencing was applied to subset of baseline samples of VS patients (ie, patients with HBV DNA <60 IU/mL at screening and baseline HBV RNA ≥3.0 $\log_{10}$ copies/mL).

Table S2: Proportion of VS Patients with VR and Nucleotide Substitutions Observed at ≥1 S-trigger Target Region POI at Time of VR and During Aggregated Off-treatment Follow-up Period - by Study and Treatment

	REEF-1 JNJ-3989		REEF-1 JNJ-3989+JNJ-6379		REEF-2 JNJ-3989+JNJ-6379		Total JNJ-3989 (±JNJ-6379)		REEF-2 NA-control arm	
VS patients, mITT, n	176		61		84		321		44	
Patients who stopped all treatment (including NA), n	39		11		77		127		40	
Patients with VR, n (%)	17 (43.6)		5 (45.5)		53 (68.8)		75 (59.1)		34 (85)	
Patients with HBV DNA >200 IU/mL at single off-treatment time point, n (%)	4 (10.3)		4 (36.4)		1 (1.3)		9 (7.1)		5 (12.5)	
Time point of viral sequencing	VR+12w†	Aggregated FU‡	VR+12w†	Aggregated FU‡	VR+12w†	Aggregated FU‡	VR+12w†	Aggregated FU‡	VR+12w†	Aggregated FU‡
Patients with S-gene sequence info available, n	17	21	5	8	48	49	70	78	33	36

No substitutions at S-trigger POI 2-18, n (%)	14 (82.4)	13 (61.9)	5 (100)	7 (87.5)	34 (70.8)	33 (67.3)	53 (75.7)	53 (67.9)	28 (84.8)	29 (80.6)
≥1 substitutions at S-trigger POI 2-18, n (%)*	3 (17.6)	8 (38.1)	0 (0.0)	1 (12.5)	14 (29.2)	16 (32.7)	17 (24.3)	25 (32.1)	5 (15.2)	7 (19.4)
264	-	-	-	-	1 (2.1)	1 (2.0)	1 (1.4)	1 (1.3)	-	-
C264C/T	-	-	-	-	1 (2.1)		1 (1.4)	-	-	-
273	2 (11.8)	6 (28.6)	-	1 (12.5)	14 (29.2)	15 (30.6)	16 (22.9)	22 (28.2)	5 (15.2)	7 (19.4)
A273A/G	-	1 (4.8)	-	-	5 (10.4)	6 (12.2)	5 (7.1)	7 (9.0)	1 (3.0)	2 (5.6)
A273G	2 (11.8)	5 (23.8)	-	1 (12.5)	9 (18.8)	13 (26.5)	11 (15.7)	19 (24.4)	4 (12.1)	5 (13.9)
276	1 (5.9)	2 (9.5)	-	-	-	1 (2.0)	1 (1.4)	3 (3.8)	-	1 (2.8)
T276T/C	-	1 (4.8)	-	-	-	1 (2.0)	-	2 (2.6)	-	1 (2.8)
T276C	1 (5.9)	1 (4.8)	-	-	-	-	1 (1.4)	1 (1.3)	-	-

mITT, modified intent-to-treat. NA, nucleos(t)ide analogue. nt, nucleotide. POI, position of interest. VR, viral relapse (ie confirmed off-treatment increase in HBV DNA >200 IU/mL in patients with HBV DNA <LLOQ at EOT). VS, virologically suppressed. FU, follow-up.

Observed variants are defined as changes from the universal HBV genotype A (NCBI ID X02763) reference sequence and if sequence read frequency was >15%. Viral sequencing data included in the analysis was considering either 1) the first assessment up to and including 12-weeks after viral flare was identified (†VR+12w), or 2) all off-treatment time points with sequencing information available (‡Aggregated FU).

*A single patient could have 1 or more nucleotide substitutions observed at 1 or more S- or X-trigger POI during aggregated off-treatment follow-up period.

Table S3: Proportion of VS Patients with VR and Nucleotide Substitutions Observed at ≥1 X-trigger Target Region POI at Time of VR and During Aggregated Off-treatment Follow-up Period - by Study and Treatment

	REEF-1 JNJ-3989		REEF-1 JNJ-3989+JNJ-6379		REEF-2 JNJ-3989+JNJ-6379		Total JNJ-3989(±JNJ-6379)		REEF-2 NA-control arm	
VS patients, mITT	176		61		84		321		44	
Patients who stopped all treatment (including NA), n	39		11		77†		127		40†	
Patients with VR, n (%)	17 (43.6)		5 (45.5)		53 (68.8)		75 (59.1)		34 (85)	
Patients with HBV DNA >200 IU/mL at single off-treatment time point, n (%)	4 (10.3)		4 (36.4)		1 (1.3)		9 (7.1)		5 (12.5)	
Time point of viral sequencing	VR+12w†	Aggregated FU‡	VR+12w†	Aggregated FU‡	VR+12w†	Aggregated FU‡	VR+12w†	Aggregated FU‡	VR+12w†	Aggregated FU‡
Patients with X-gene sequence info available, n	17	21	4	7	48	49	69	77	33	35
No substitutions at X-trigger POI 2-18, n (%)	13 (76.5)	12 (57.1)	4 (100)	4 (57.1)	31 (64.6)	18 (36.7)	48 (69.6)	34 (44.2)	31 (94.0)	33 (94.3)
≥1 substitutions at X-trigger POI 2-18, n (%)*	4 (23.5)	9 (42.9)	0 (0.0)	3 (42.9)	17 (35.4)	31 (63.3)	21 (30.4)	43 (55.8)	2 (6.0)	2 (5.7)
1784	1 (5.9)	3 (14.3)	-	1 (14.3)	1 (2.1)	1 (2.0)	2 (2.9)	5 (6.5)	-	-
T1784T/C	1 (5.9)	1 (4.8)	-	-	-	1 (2.0)	1 (1.4)	3 (3.9)	-	-
T1784C		3 (14.3)	-	1 (14.3)	1 (2.1)	1 (2.0)	1 (1.4)	4 (5.2)	-	-
1785	2 (11.8)	3 (14.3)	-	-	2 (4.2)	11 (22.4)	4 (5.8)	14 (18.2)	-	-
A1785A/C	1 (5.9)	1 (4.8)	-	-	-	1 (2.0)	1 (1.4)	2 (2.6)	-	-

	REEF-1 JNJ-3989		REEF-1 JNJ-3989+JNJ-6379		REEF-2 JNJ-3989+JNJ-6379		Total JNJ-3989(±JNJ-6379)		REEF-2 NA-control arm	
A1785C	-	-	-	-	-	4 (8.2)	-	4 (5.2)	-	-
A1785A/G	-	-	-	-	1 (2.1)	5 (10.2)	1 (1.4)	5 (6.5)	-	-
A1785A/T	-	1 (4.8)	-	-	-	1 (2.0)	-	2 (2.6)	-	-
A1785C/T	-	-	-	-	-	1 (2.0)	-	1 (1.3)	-	-
A1785T	1 (5.9)	1 (4.8)	-	-	1 (2.1)	1 (2.0)	2 (2.9)	2 (2.6)	-	-
1787	-	-	-	-	-	1 (2.0)	-	1 (1.3)	-	-
G1787A	-	-	-	-	-	1 (2.0)	-	1 (1.3)	-	-
1793	1 (5.9)	1 (4.8)	-	-	-	-	1 (1.4)	1 (1.3)	-	-
A1793G	1 (5.9	1 (4.8)	-	-	-	-	1 (1.4)	1 (1.3)	-	-
1794	1 (5.9)	4 (19.0)	-	2 (28.6)	14 (29.2)	27 (55.1)	15 (21.7)	33 (42.9)	1 (3.0)	1 (2.9)
T1794T/A	1 (5.9)	3 (14.3)	-	-	6 (12.5)	17 (34.7)	7 (10.1)	20 (26.0)	-	-
T1794A	-	1 (4.8)	-	1 (14.3)	8 (16.7)	19 (38.8)	8 (11.6)	21 (27.3)	1 (3.0)	1 (2.9)
T1794T/G	-	1 (4.8)	-	1 (14.3)	1 (2.1)	3 (6.1)	1 (1.4)	5 (6.5)	-	-
T1794G	-	1 (4.8)	-	-	-	2 (4.1)	-	3 (3.9)	-	-
1795	-	1 (4.8)	-	-	-	3 (6.1)	-	4 (5.2)	-	-
T1795T/G	-	-	-	-	-	2 (4.1)	-	2 (2.6)	-	-
T1795G	-	1 (4.8)	-	-	-	1 (2.0)	-	2 (2.6)	-	-
1796	-	-	-	-	-	1 (2.0)	-	1 (1.3)	-	-
G1796G/A	-	-	-	-	-	1 (2.0)	-	1 (1.3)	-	-
1797	-	-	-	-	-	-	-	-	1 (3.0)	1 (2.9)
G1797G/A	-	-	-	-	-	-	-	-	1 (3.0)	1 (2.9)
G1797A	-	-	-	-	-	-	-	-	1 (3.0)	1 (2.9)

EOT, end of treatment. HBV, hepatitis B virus. mITT, modified intent-to-treat. NA, nucleos(t)ide analogue. nt, nucleotide. POI, position of interest. VR, viral relapse (ie, confirmed off-treatment increase in HBV DNA >200 IU/mL in patients with HBV DNA <LLOQ at EOT). VS, virologically suppressed. FU, follow-up.

	REEF-1 JNJ-3989	REEF-1 JNJ-3989+JNJ-6379	REEF-2 JNJ-3989+JNJ-6379	Total JNJ-3989(±JNJ-6379)	REEF-2 NA-control arm

Observed variants are defined as changes from the universal HBV genotype A (NCBI ID X02763) reference sequence and if sequence read frequency was >15%.

Viral sequencing data included in the analysis was considering either 1) the first assessment up to and including 12-weeks after viral flare was identified (†VR+12w), or 2) all off-treatment time points with sequencing information available (‡Aggregated FU).

*A single patient could have 1 or more nucleotide substitutions observed at 1 or more S- or X-trigger POI during aggregated off-treatment follow-up period.

†In REEF-2, 7/84 active arm and 4/44 control arm patients either did not stop NA or discontinued study treatment early (2).

Table S4: Median (Range) Time from End-of-Treatment to VR in VS HBeAg-negative patients (REEF-2) - by Treatment and Presence of S- (Top) and X- (Bottom) Trigger Target Region nt Substitutions

Median (range) Time to VR, days	ETV as backbone NA	TDF/TAF as backbone NA
PBO+NA	112 (54-196; N=14)	29 (15-56; N=20)
JNJ-3989+JNJ-6379+NA	144 (57-257; N=17)	153 (28-337; N=36)
with X substitution	140 (57-257; n=10)	141 (28-309; n=21)
without X substitution	169 (140-212; n=6)	139 (56-337; n=12)
with S substitution	126 (57-207; n=6)	167 (28-309; n=10)
without S substitution	156 (115-257; n=10)	113 (28-337; n=23)
No sequence info available*	224 (ND; n=1)	296 (295-337; n=3)

NA, nucleos(t)ide analogue. PBO, placebo. EOT, end of treatment. ETV, entecavir. VR, viral relapse. VS, virologically suppressed. TDF/TAF, tenofovir. ND, not determinable.

N, number of patients with VR (ie, confirmed off-treatment HBV DNA >200 IU/mL).

n, number of patients with VR and with off-treatment sequence data available. *Sequencing of off-treatment samples failed for 4 JNJ-3989-treated patients with VR.

Time to VR was calculated relative to end-of-treatment visit and was considered the first off-treatment time point with HBV DNA >200 IU/mL.

The S- and X-trigger target region positions of interest (POI) within the S- and X-genes were nt261-278 and nt1781-1798 (HBV full genome numbering based on EcoRI restriction site), respectively.

Table S5: Prevalence Among HBV Sequences in Public Database

Variant	Prevalence
S: A273G	771/17795 (4.3%)
X: T1794A	10/5947 (0.17%)
X: T1794G	4/5947 (0.07%)
X: T1795G	10/5947 (0.17%)
X: T1784C	7/5947 (0.12%)
X: A1785C	6/5947 (0.10%)
X: A1785G	0/5947 (0.0%)
X: A1785T	13/5947 (0.22%)

HBV, hepatitis B virus.

HBVdb public HBV sequence database can be accessed via https://hbvdb.ibcp.fr/HBVdb/ (HBVdb export of sequences included in this report was performed in April, 2016) (3)

Supplementary figures

Fig. S1: REEF-1 study design (4).

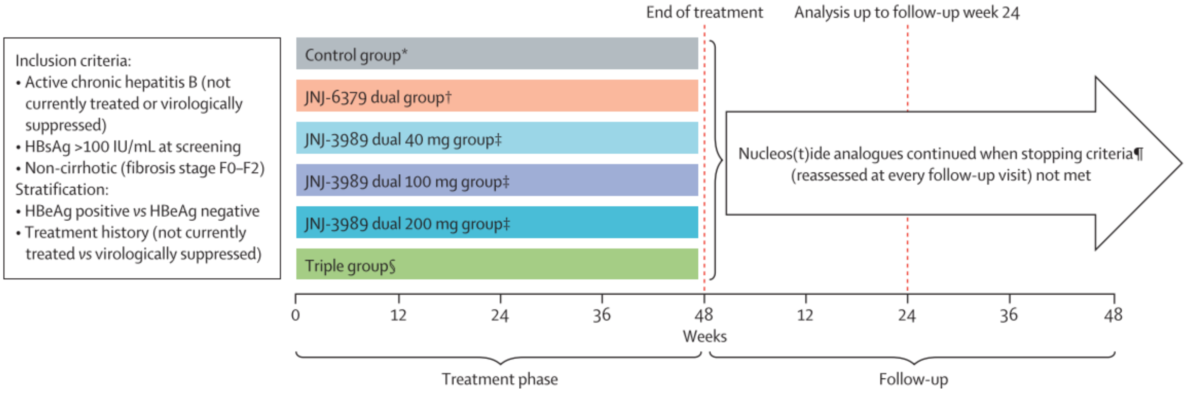

HBeAg, hepatitis B e antigen.

Fig. S2: REEF-2 study design (2).

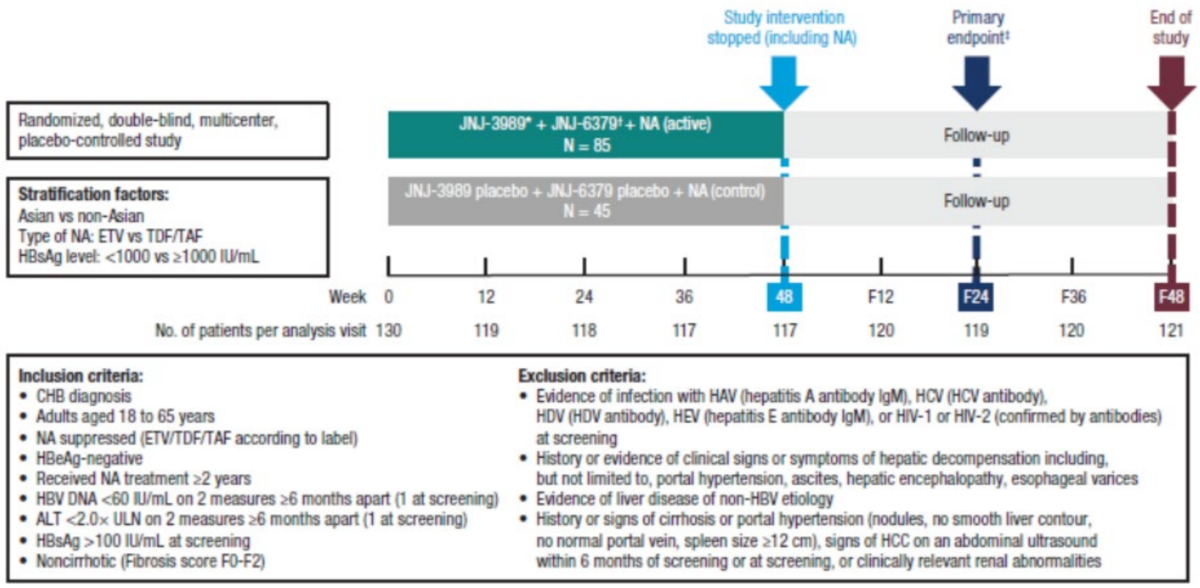

ALT, alanine transaminase; CHB, chronic hepatitis B; ETV, entecavir; F, follow-up; HAV, hepatitis A virus; HBeAg, hepatitis B e antigen; HBsAg, hepatitis B surface antigen; HCC, hepatocellular carcinoma; HCV, hepatitis C virus; HDV, hepatitis D virus, HEV, hepatitis E virus; HIV, human immunodeficiency virus; JNJ-3989, JNJ-73763989; JNJ-6379, JNJ-56136379; LLOQ, lower limit of quantification; NA, nucleos(t)ide analog; PO, oral; SC, subcutaneous; TAF, tenofovir alafenamide; TDF, tenofovir disoproxil fumarate; ULN, upper limit of normal.

*200 mg SC every 4 weeks.

†250 mg PO daily.

‡HBsAg seroclearance (HBsAg <LLOQ [0.05 IU/mL]) at Week 72 without restarting NA treatment.

Fig. S3: HBV genomic organization and sequencing strategies.

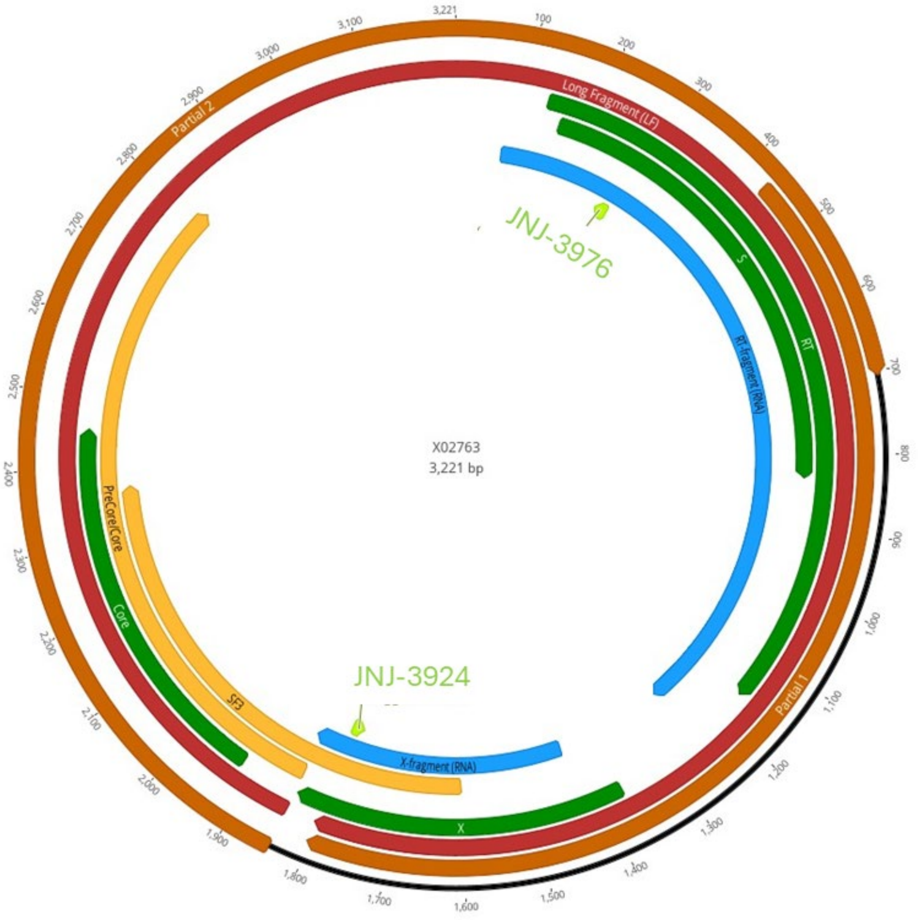

HBV genome sequence shown is the universal HBV genotype A reference sequence (NCBI ID X02763). HBV genetic regions of interest HBV core, RT-domain, S- and X-gene are shown in green. Nucleotide numbering based on EcoR1 restriction site.

Main and back-up HBV full genome sequencing approaches are shown in red (long fragment [LF]) and orange (BU LF with partial 1 and partial 2 overlapping fragments). Exploratory HBV DNA based sub-genomic sequencing approaches are shown in yellow (i.e., PreCore/Core and Short fragment 3 [SF3)]. Exploratory HBV RNA based sub-genomic sequence approaches are shown in blue (i.e., RT- and X-fragment).

JNJ-3976 ("S-trigger") and JNJ-3924 ("X-trigger") trigger target regions (nt261-268 and nt1781-1798, respectively) are indicated in light green highlight.

Alignment was generated using Geneious Prime.

Fig. S4: (a) Schematic representation of HBV RNA RT-qPCR and HBV RNA RT-domain amplification strategies and (b) Phylogenetic tree based on the 25% consensus sequences of the obtained (n = 23) HBV RNA genotypes (including the reference sequences from genotype A-I).

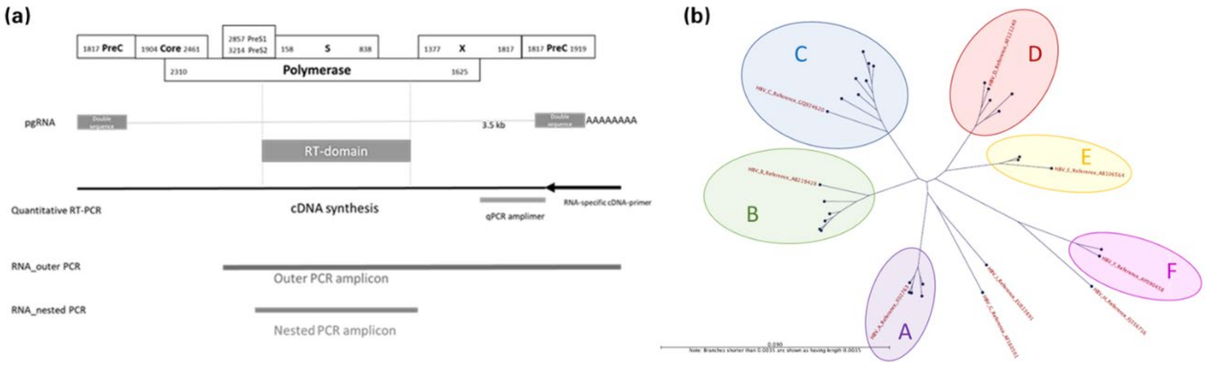

Fig. S5: Representative examples of VS, JNJ-3989-treated patients (all REEF-2) with VR and X-trigger region nt substitution(s) observed during off-treatment follow-up.

Patient 1

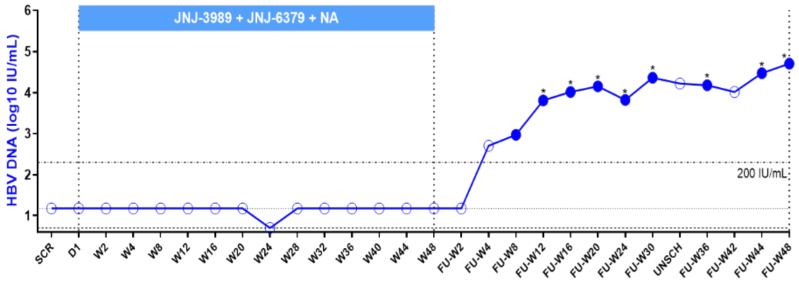

Substitution, %*	FU W8	FU W12	FU W16	FU W20	FU W24	FU W30	FU W36	FU W44	FU W48
**T1794A**	-	70.02	79.71	78.52	99.91	76.22	99.86	99.80	88.60

Patient 2

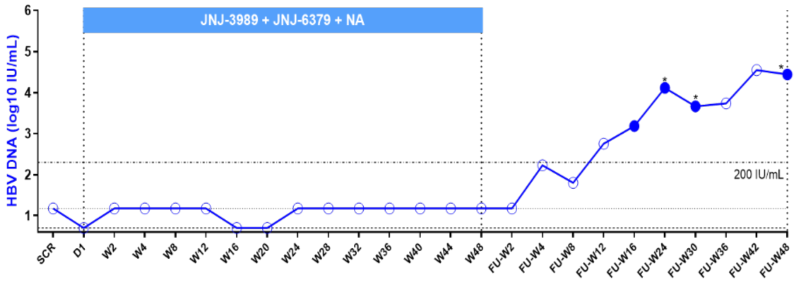

Substitution, %*	FU W16	FU W24	FU W30	FU W48
**T1794A**	6.89	51.98	85.76	80.99

Patient 3

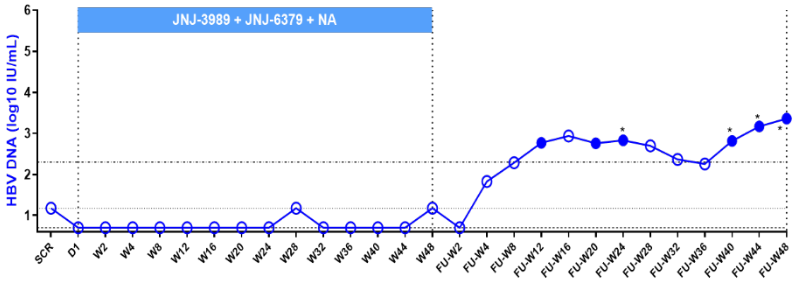

Substitution, %*	FU W12	FU W20	FU W24	FU W40	FU W44	FU W48
**T1794A**	4.74	6.84	99.86	99.86	99.79	99.86

Patient 4

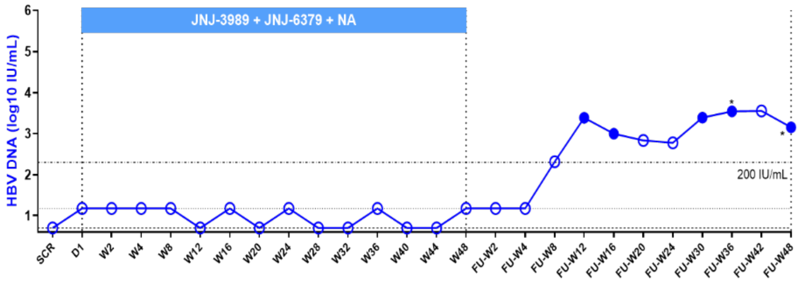

Substitution, %*	FU W12	FU W16	FU W30	FU W36	FU W48
**T1794A**	-	-	-	41.56	73.68

SCR, screening. D, day. FU-W, follow-up Week. NA, nucleos(t)ide analogue. “-”, sequenced but no variant observed.

Closed and open dots indicate visits with and without HBV sequence information available, respectively. Asterix (*) represents visit at which the nt substitution was observed with sequence-read frequency >15%.

*X-trigger region nt substitution of interest and Illumina MiSeq sequencing sequence read-frequencies (%) are shown. Wild type nt variant is not shown.

Supplementary references

1. FDA. Submitting next generation sequencing data to the division of antiviral products. In: US Depertment of Health and Human Services FaDAF, Center for Drug Evaluation and Research (CDER), editor. https://www.fda.gov/regulatory-information/search-fda-guidance-documents/submitting-next-generation-sequencing-data-division-antiviral-products-guidance-industry-technical2019.

2. Agarwal K, Buti M, van Bommel F, et al. JNJ-73763989 and bersacapavir treatment in nucleos(t)ide analogue-suppressed patients with chronic hepatitis B: REEF-2. J Hepatol. 2024;81(3):404-14.

3. Hayer J, Jadeau F, et al. HBVdb: a knowledge database for Hepatitis B Virus. Nucleic Acids Res. 2013 Jan;41.

4. Yuen MF, Asselah T, Jacobson IM, et al. Efficacy and safety of the siRNA JNJ-73763989 and the capsid assembly modulator JNJ-56136379 (bersacapavir) with nucleos(t)ide analogues for the treatment of chronic hepatitis B virus infection (REEF-1): a multicentre, double-blind, active-controlled, randomised, phase 2b trial. Lancet Gastroenterol Hepatol. 2023;8(9):790-802.
