# Supplementary material for: Viral sequence analysis of chronic hepatitis B patients treated with the siRNA JNJ-73763989 in phase II clinical trials
Source: JHEP Rep. 2025 Oct 9;7(12):101618. doi: 10.1016/j.jhepr.2025.101618 (PMC12682118; doi:10.1016/j.jhepr.2025.101618)
Supplement: Multimedia component 2 [file mmc2.docx]

**JHEP Reports**

**CTAT methods**

Tables for a “Complete, Transparent, Accurate and Timely account” (CTAT) are now mandatory for all revised submissions. The aim is to enhance the reproducibility of methods.

- Only include the parts relevant to your study
- Refer to the CTAT in the main text as ‘Supplementary CTAT Table’
- Do not add subheadings
- Add as many rows as needed to include all information
- Only include one item per row

**If the CTAT form is not relevant to your study, please outline the reasons why:**

|  |
| --- |

- 1. **Antibodies**

| **Name** | **Citation** | **Supplier** | **Cat no.** | **Clone no.** |
| --- | --- | --- | --- | --- |
| **Not applicable** |  |  |  |  |

- 1. **Cell lines**

| **Name** | **Citation** | **Supplier** | **Cat no.** | **Passage no.** | **Authentication test method** |
| --- | --- | --- | --- | --- | --- |
| **Not applicable** |  |  |  |  |  |

- 1. **Organisms**

| **Name** | **Citation** | **Supplier** | **Strain** | **Sex** | **Age** | **Overall n number** |
| --- | --- | --- | --- | --- | --- | --- |
| **Not applicable** |  |  |  |  |  |  |

- 1. **Sequence based reagents**

| **Name** | **Sequence** | **Supplier** |
| --- | --- | --- |
| **Not applicable** |  |  |

- 1. **Biological samples**

| **Description** | **Source** | **Identifier** |
| --- | --- | --- |
| **Not applicable** |  |  |

- 1. **Deposited data**

| **Name of repository** | **Identifier** | **Link** |
| --- | --- | --- |
| Yale Open Data Access (YODA) Project |  | <http://yoda.yale.edu> |

- 1. **Software**

| **Software name** | **Manufacturer** | **Version** |
| --- | --- | --- |
| **Not applicable** |  |  |

- 1. **Other (*e.g*. drugs, proteins, vectors etc.)**

| siRNA JNJ-73763989 |  |  |
| --- | --- | --- |
| CAM-E JNJ-56136379 |  |  |
| Nucleos(t)ide analogs (ETV, TAF, TDF) |  |  |

- 1. **Please provide the details of the corresponding methods author for the manuscript:**

| **Thierry Verbinnen, Johnson & Johnson innovative Medicine** |
| --- |

**2.0 Please confirm for randomised controlled trials all versions of the clinical protocol are included in the submission. These will be published online as supplementary information.**

| **This paper describes a secondary analysis of 2 phase studies already described before. All study related materials and documents have already been included in the below references.**   - Agarwal K, Buti M, van Bommel F, Lampertico P, Janczewska E, Bourliere M, et al. JNJ-73763989 and bersacapavir treatment in nucleos(t)ide analogue-suppressed patients with chronic hepatitis B: REEF-2. J Hepatol. 2024;81(3):404-14. - Yuen MF, Asselah T, Jacobson IM, Brunetto MR, Janssen HLA, Takehara T, et al. Efficacy and safety of the siRNA JNJ-73763989 and the capsid assembly modulator JNJ-56136379 (bersacapavir) with nucleos(t)ide analogues for the treatment of chronic hepatitis B virus infection (REEF-1): a multicentre, double-blind, active-controlled, randomised, phase 2b trial. Lancet Gastroenterol Hepatol. 2023;8(9):790-802 |
| --- |
