# Supplementary material for: Viral sequence analysis of chronic hepatitis B patients treated with the siRNA JNJ-73763989 in phase II clinical trials
Source: JHEP Rep. 2025 Oct 9;7(12):101618. doi: 10.1016/j.jhepr.2025.101618 (PMC12682118; doi:10.1016/j.jhepr.2025.101618)
Supplement: Multimedia component 4 [file mmc4.pdf]

Viral sequence analysis of chronic hepatitis B patients treated with the siRNA JNJ-73763989 in phase II clinical trials

Authors

Thierry Verbinnen, Erkki Lathouwers, John Jezorwski, …, Man-Fung Yuen, Sandra De Meyer, Oliver Lenz

Correspondence

tverbinn@its.jnj.com (T. Verbinnen).

Graphical abstract

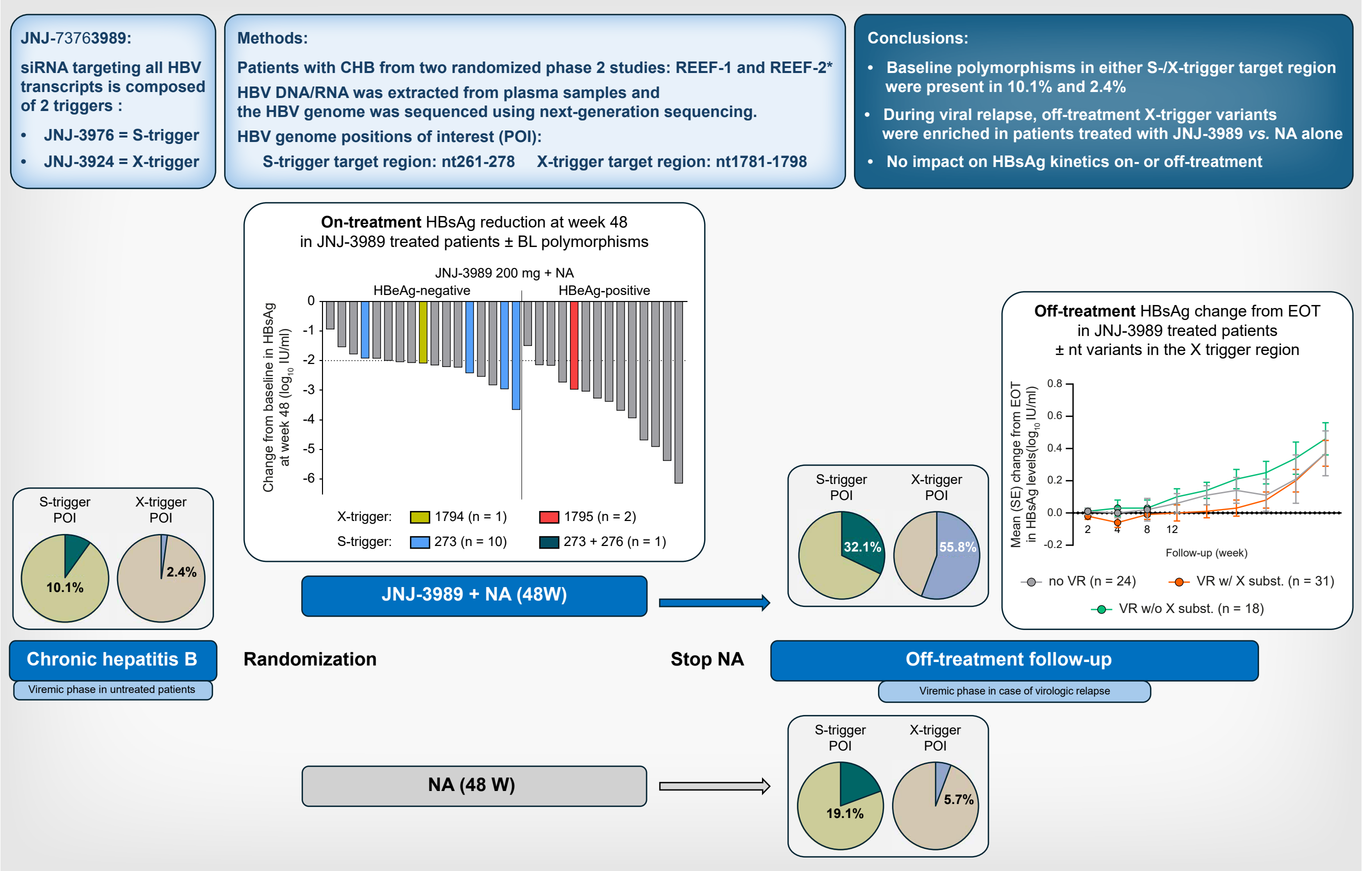

Highlights:

- JNJ-3989 S- and X-trigger target region baseline polymorphisms were present in 10.1% and 2.4% of patients with CHB.
- No impact of these baseline polymorphisms on JNJ-3989 induced HBsAg and HBeAg declines.
- JNJ-3989-treated patients with viral relapse frequently had substitutions within the X-trigger target region.
- These X-trigger substitutions likely emerged following increased HBV replication after stopping all treatment.
- The detection of S-and X-trigger substitutions was not associated with differences in off-treatment HBsAg kinetics.

https://doi.org/10.1016/j.jhepr.2025.101618

© 2025 The Author(s). Published by Elsevier B.V. on behalf of European Association for the Study of the Liver (EASL). This is an open access article under the CC BY license (http://creativecommons.org/licenses/by/4.0/).

Impact and implications:

Small-interfering RNA (siRNA) therapy with JNJ-3989 in patients with chronic hepatitis B induces potent, dose-dependent reductions in viral antigens, though the magnitude of decline varies among individuals. Baseline nucleotide polymorphisms in JNJ-3989 trigger target regions did not account for variability in HBsAg or HBeAg responses. Substitutions in siRNA trigger target regions were frequently detected during and after virologic relapse in JNJ-3989-treated patients, confirming antiviral target engagement. However, the emergence of these variants did not affect off-treatment HBsAg or HBV DNA kinetics. This study provides the first comprehensive clinical virology analysis of siRNA-based therapy in HBV infection, offering insights relevant to the broader development of antiviral siRNA therapeutics. The clinical significance of X-trigger substitutions for potential re-treatment with JNJ-3989 or other HBV-targeting siRNAs remains to be determined.

https://doi.org/10.1016/j.jhepr.2025.101618

Viral sequence analysis of chronic hepatitis B patients treated with the siRNA JNJ-73763989 in phase II clinical trials

Thierry Verbinnen[1,*], Erkki Lathouwers[1], John Jezorwski[2], Michael Biermer[1], Ilse Augustyns[1], Craig Grant[3], Kosh Agarwal[4], Man-Fung Yuen[5], Sandra De Meyer[1], Oliver Lenz[1]

JHEP Reports **2025**. vol. 7 | 1–6

Check for updates

Background & Aims: JNJ-73763989 (JNJ-3989) is a small-interfering RNA composed of two triggers targeting the HBsAg and HBx protein open reading frame, designed to target all HBV RNAs for degradation. JNJ-3989 + nucleos(t)ide analogue (NA) treatment dose-dependently reduced chronic hepatitis B (CHB) viral antigens. Viral sequence changes in the JNJ-3989 S-/X-trigger target regions were evaluated at baseline, on-treatment, and in patients with virologic relapse (VR) after discontinuation of all treatment in REEF-1 (NCT03982186) and REEF-2 (NCT0412954) studies.

Methods: HBV DNA/RNA was extracted from plasma samples, and the HBV genome was sequenced using next-generation sequencing. Nucleotide variants were defined as changes *vs.* the universal HBV reference sequence (read frequency >15%).

Results: Baseline polymorphisms in the JNJ-3989 target region complementary to positions 2-18 of the S-/X-trigger were present in 10.1% and 2.4% of not currently treated patients, respectively, with no relevant impact on JNJ-3989-induced HBsAg decline. Variants at X-trigger target region positions of interest (POI) were more frequently observed off treatment in JNJ-3989-treated virologically suppressed (VS) patients with VR *vs*. NA-control arm VS patients with VR (55.8% *vs*. 5.7%, respectively). Variants at S-trigger POI were observed off treatment in 32.1% and 19.4% of JNJ-3989- and NA-control treated VS patients with VR, respectively. Off-treatment HBsAg kinetics did not differ between JNJ-3989-treated patients with and without variants in S-/X-trigger target POI during VR.

Conclusion: Baseline sequence polymorphisms did not impact JNJ-3989 treatment response. JNJ-3989-treated patients who experienced VR post-treatment had variants in the X-trigger target region during VR, but not at baseline or on-treatment, suggesting that X-trigger variants developed off treatment in JNJ-3989-treated patients. The presence of these variants did not impact off-treatment HBsAg kinetics.

ClinicalTrial.gov Identifiers: NCT03982186 and NCT0412954.

© 2025 The Author(s). Published by Elsevier B.V. on behalf of European Association for the Study of the Liver (EASL). This is an open access article under the CC BY license (http://creativecommons.org/licenses/by/4.0/).

Introduction

JNJ-73763989 (JNJ-3989) is a liver-targeted small-interfering RNA (siRNA) designed to target all HBV RNAs for degradation, thereby reducing all HBV viral proteins and pregenomic RNA.[1] JNJ-3989 is composed of two triggers, JNJ-3976 (“S-trigger”) and JNJ-3924 (“X-trigger”), both synthetic, 21-nucleotide (nt) long, double-stranded siRNA molecules targeting the hepatitis B surface antigen (HBsAg) and hepatitis B x protein open reading frames, respectively. The phase IIb REEF-1 (NCT03982186) and REEF-2 (NCT0412954) studies showed that JNJ-3989-based treatment was well tolerated by patients with chronic hepatitis B (CHB) and led to JNJ-3989 dose-dependent reductions in all viral markers.[2,3]

Sequence-specific siRNAs, like JNJ-3989, contain a 5’ (nucleotide position 2-8) seed region critical for target mRNA recognition, and a 3’ supplemental region for binding stability and specificity. Effective silencing requires a (near-)perfect match between seed region and RNA target.[4–7] Pre-existing and emerging viral resistance to direct antiviral drugs, resulting in lack of activity and subsequent treatment failure, is well documented.[8–10] Our understanding of viral resistance to viral genome targeting siRNAs is mainly restricted to *in vitro* data, given limited experience with this type of agent in the clinic.[11–13]

Here we assessed viral sequence changes within the S-/X-trigger target regions in HBV-infected patients enrolled in REEF-1 and REEF-2, focusing on viral sequence variants present either prior to the start of JNJ-3989 treatment or observed in post-baseline samples from patients with virologic relapse. In addition, the impact of these viral sequence substitutions on response to JNJ-3989 treatment was evaluated.

Patients and methods

Study design and patients

REEF-1[3] (Fig S1) and REEF-2[2] (Fig S2) were randomized, double-blind, multi-center, placebo-controlled studies of

* Corresponding author. Address: Thierry Verbinnen, Turnhoutseweg 30, 2340, Beerse, Belgium; Tel.: +3214641786.
E-mail address: tverbinn@its.jnj.com (T. Verbinnen).
https://doi.org/10.1016/j.jhepr.2025.101618

patients with CHB who received 48 weeks of treatment with JNJ-3989/placebo + NA (daily; oral) followed by 48 weeks of follow-up. Patients in REEF-1 were eligible to stop NA treatment if they met pre-defined NA stopping criteria (HBsAg <10 IU/ml, HBV DNA <LLOQ (lower limit of quantification), hepatitis B e antigen (HBeAg)-negative, alanine aminotransferase [ALT] <3 × the upper limit of normal) at Week 48 or during 48-weeks follow-up. Patients in REEF-2, all virologically suppressed (VS) at baseline, stopped NA treatment at Week 48. Patients stopping all treatment were monitored for pre-defined NA re-treatment criteria based on increases in HBV DNA and/or ALT.

Both studies were conducted in full compliance with the Declaration of Helsinki and Good Clinical Practice guidelines. All patients provided written informed consent.

Viral sequence analysis

HBV DNA-based full genome sequencing

HBV DNA was extracted from plasma samples with sufficiently high viral DNA levels, and the full HBV DNA genome was sequenced using Illumina MiSeq sequencing (Illumina, San Diego, CA, USA) with a 1% sequence read cut-off (supplementary methods).[14]

Viral sequence analyses focused on nt changes in the HBV target regions complementary to positions 2-18 of the JNJ-3989 S- (nt261-278) and X-trigger (nt1781-1798), which are considered potentially relevant for siRNA target binding and activity.[15,16]

Exploratory HBV RNA-based sequencing

Baseline samples from REEF-1 VS patients and a subset of Week 48 samples from REEF-1 not currently treated (NCT) patients were sequenced using plasma HBV RNA. The HBV RNA reverse-transcription PCR used for sequencing was based on a previously described assay aimed to detect the polyadenylated 3'end of all covalently closed circular DNA-derived HBV RNA transcripts, including pregenomic RNA (supplementary methods).[17]

Results

Baseline polymorphisms and impact on serologic response

Baseline polymorphisms in the target region complementary to positions 2-18 of the JNJ-3989 S-/X-triggers were present in 17/168 (10.1%) and 4/165 (2.4%) NCT REEF-1 patients, respectively (Table S1). Most patients had single nt polymorphisms at S-trigger position of interest (POI) 273, and two each had a single nt polymorphism at X-trigger POI 1794 or 1795. One patient had a combination of nt polymorphisms at S-trigger POI 273 and 276.

Due to low or negative HBV DNA levels in VS patients, baseline samples of REEF-1 VS patients were sequenced using plasma HBV RNA as starting material. Five of 36 (13.9%) and 0/41 (0.0%) REEF-1 VS patients with baseline HBV RNA-based sequence data available had polymorphisms at S-/X-trigger POI, respectively; four had a single nt polymorphism (A273G) and one had A273G+T276 C/T polymorphisms (Table S1).

Comparison of the individual HBe/sAg declines from baseline for patients with and without baseline polymorphisms confirmed that baseline sequence polymorphisms in the siRNA target regions did not impact serologic response to JNJ-3989-based treatment (Fig. 1A).

Viral sequencing analysis of patients with VR

Post-baseline HBV DNA-based S-/X-gene sequence data were available from >90% of VS patients with VR (Table 1). Among VS patients with VR, X-trigger target region nt substitutions were more frequently observed during off-treatment follow-up visits in JNJ-3989 ± JNJ-6379-treated patients than in NA-treated patients (55.8% and 5.7%, respectively; Table 1). More specifically, nt substitutions were observed at X-trigger target POI 1784, 1785, 1794, and 1795 at a range of 5.2-42.9% among JNJ-3989 ± JNJ-6379-treated VS patients with VR and at a range of 0.0-2.9% among NA-only-treated VS patients with VR (Tables 1 and S2). S-trigger target region nt substitutions were observed during off-treatment follow-up visits in 32.1% of JNJ-3989-treated VS patients and 19.4% of NA-only-treated VS patients with VR (Tables 1 and S3). Most had substitutions at S-trigger POI 273.

Exploratory HBV RNA-based sequencing of on-treatment week 48 samples

To evaluate if S-/X-trigger target region variants were already emerging during JNJ-3989 treatment, Week 48 samples from JNJ-3989-treated REEF-1 NCT patients were analyzed. Because most NCT patients achieve HBV DNA <LLOQ early on-treatment,[3] HBV RNA-based sequencing was performed on Week 48 plasma samples.

Week 48 S- and X-gene regions were successfully sequenced for 25/30 (83.3%) and 28/30 (93.3%) patients, respectively, who had a Week 48 HBV RNA level of ~3.0 $\log_{10}$ copies/ml or higher (data on file). None of these patients with paired baseline (HBV DNA-based) and Week 48 (HBV RNA-based) sequence data had emerging substitutions at any of the S-/X-trigger POI.

Off-treatment response in REEF-2 patients with S- and/or X-trigger target region variants observed during VR

There were no differences in median time to VR between JNJ-3989-treated patients with or without S-/X-trigger target region substitutions (Fig. 1B, Table S4).

Among JNJ-3989-treated patients, S-/X-trigger target region variants were generally detected at similar frequency in those reaching high or low peak HBV DNA values during VR (Fig. 1C). All three active arm patients with biochemical flare (including one patient with peak HDV DNA >100,000 IU/ml) had X-trigger but no S-trigger target region substitutions during VR (data on file).

There was no relevant difference between off-treatment HBsAg kinetics of JNJ-3989-treated patients with or without VR, and between patients with or without S-/X-trigger target region substitutions observed during or after VR (Fig. 1D).

Discussion

JNJ-3989-based treatment led to potent and dose-dependent declines in viral antigens in patients with CHB.[2,3] However,

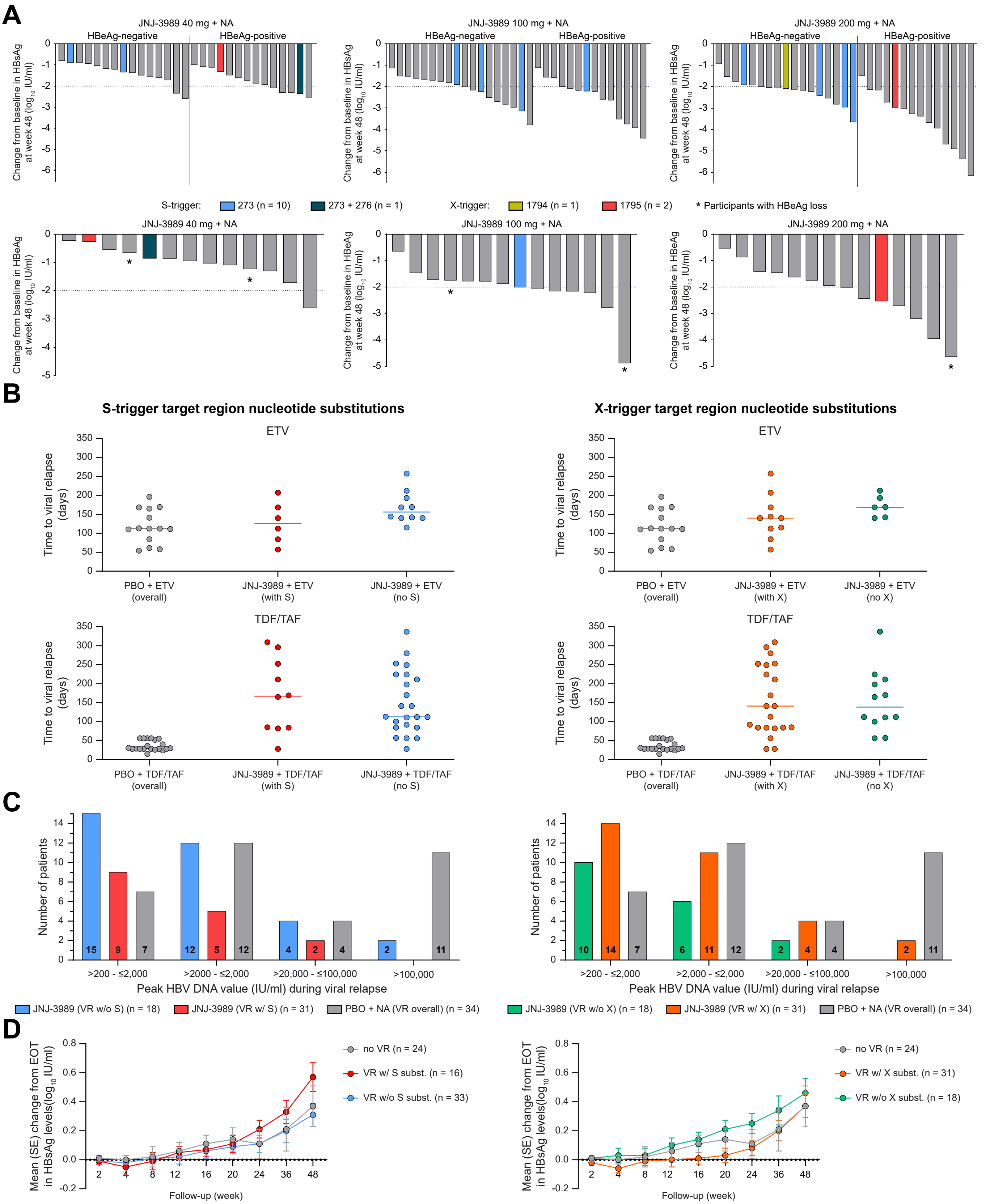

Fig. 1. HBV antigen responses and HBV kinetics by presence of S- and X-trigger target region nucleotide substitutions. (A) Only NCT participants who completed 48 weeks of JNJ-3989 + NA treatment and had S- and X-gene sequence data available at baseline. (B) Patients with VR (*i.e.*, confirmed off-treatment HBV DNA >200 IU/ml) and off-treatment sequence data available. (C) Peak HBV DNA category (*i.e.*, >200, >2,000, >20,000, and >100,000 IU/ml) had to be confirmed at ≥2

Table 1. Proportion of VS patients with VR and nucleotide substitutions observed at ≥1 S- and X-trigger target region POI at time of VR and during aggregated off-treatment follow-up period – by study and treatment.

	Total REEF-1 & REEF-2 JNJ-3989 (±JNJ-6379) + NA		REEF-2 NA-control arm	
VS patients, mITT, n		321		44
Patients who stopped all treatment (including NA), n		127		40
Patients with VR, n (%)		75 (59.1)		34 (85)
Patients with HBV DNA >200 IU/ml at single off-treatment time point, n (%)		9 (7.1)		5 (12.5)
**Time point of viral sequencing**	**VR + 12w†**	**Aggregated FU‡**	**VR + 12w†**	**Aggregated FU‡**
Patients with S-gene sequence info available, n	70	78	33	36
≥1 substitutions at S-trigger POI 2-18, n (%)*	17 (24.3)	25 (32.1)	5 (15.2)	7 (19.4)
264	1 (1.4)	1 (1.3)	-	-
273	16 (22.9)	22 (28.2)	5 (15.2)	7 (19.4)
276	1 (1.4)	3 (3.8)	-	1 (2.8)
Patients with X-gene sequence info available, n	69	77	33	35
≥1 substitutions at X-trigger POI 2-18, n (%)*	21 (30.4)	43 (55.8)	2 (6.0)	2 (5.7)
1784	2 (2.9)	5 (6.5)	-	-
1785	4 (5.8)	14 (18.2)	-	-
1787	-	1 (1.3)	-	-
1793	1 (1.4)	1 (1.3)	-	-
1794	15 (21.7)	33 (42.9)	1 (3.0)	1 (2.9)
1795	-	4 (5.2)	-	-
1796	-	1 (1.3)	-	-
1797	-	-	1 (3.0)	1 (2.9)

EOT, end of treatment; FU, follow-up; mITT, modified intent-to-treat; NA, nucleos(t)ide analogue; nt, nucleotide; POI, position of interest; VR, viral relapse (*i.e.*, confirmed off-treatment increase in HBV DNA >200 IU/ml in patients with HBV DNA <LLOQ at EOT); VS, virologically suppressed.
Observed variants are defined as changes from the universal HBV genotype A (NCBI ID X02763) reference sequence with sequence read frequency was >15%.
Viral sequencing data included in the analysis was considering either i) the first assessment up to and including 12 weeks after viral flare was identified (†VR+12w), or ii) all off-treatment time points with sequencing information available (‡Aggregated FU).
*A single patient could have one or more nucleotide substitutions observed at one or more S- or X-trigger POI during the aggregated off-treatment follow-up period.
†In REEF-2, 7/84 active arm and 4/44 control arm patients either did not stop NA or discontinued study treatment early.[6]

substantial inter-patient variability in the magnitude of antigen declines was observed. After stopping JNJ-3989 treatment, HBsAg levels increased in most patients but with different kinetics, and with a relevant proportion of patients maintaining sustained suppressed HBsAg levels.[2,18] Here, we aimed to determine if pre-existing viral sequence substitutions and post-baseline sequence changes influenced response to JNJ-3989 treatment. Our study represents the first comprehensive clinical virology analysis for siRNA-based therapy in HBV and other viral diseases.

Baseline polymorphisms in the siRNA target regions were infrequent, and HBsAg declines in NCT patients with these variants were comparable to those without variants, suggesting no relevant effect of these sequence variants on JNJ-3989 activity.

Viral sequencing at time of viral relapse showed that most JNJ-3989-treated patients with VR had substitutions at positions complementary to siRNA antisense positions in either the seed (1794 and 1795) or supplemental region (1784 and 1785) of the X-trigger siRNA. As most patients who discontinued NA treatment and were therefore at risk of VR were VS due to long-term NA therapy at study entry, assessment of whether X-trigger substitutions emerged post-baseline could only be made indirectly. Based on indirect comparison of baseline and available Week 48 sequences, as well as public sequence databases (Table S5), it can be concluded that these variants emerged off treatment secondary to HBV DNA increases in JNJ-3989-treated patients who stopped all treatment (see Fig. S5 for representative examples). S-trigger target-region variants were observed at similar frequency in baseline samples of NCT patients, and in samples of JNJ-3989-treated and NA-only treated patients with VR, suggesting S-trigger variants were not newly emerging in patients with VR. The reason for the difference in the emergence of S-/X-trigger target region variants might be due to a lower barrier to resistance of the X-gene region, coding for the HBV X protein only, whereas the S-gene region codes for multiple HBV proteins.[19] Intrahepatic samples from JNJ-3989-treated patients 28-weeks after last JNJ-3989 injection showed that both S-/X-triggers were still measurable at similar concentrations.[20,21] The backbone NAs entecavir and tenofovir, as well as the CAM-E JNJ-6379 are eliminated faster than the siRNA triggers.[22–24] These subtle differences in the elimination rates between the JNJ-3989-triggers and the small molecule antiviral agents could result in a tail of low-dose JNJ-3989 monotherapy during follow-up, facilitating selection of JNJ-3989 variants. Further evaluation is needed to determine if these X-trigger target region substitutions result in reduced *in vitro* susceptibility to JNJ-3989 treatment. The lack of a suitable *in vitro* HBV system to perform resistance testing is a challenge in the field. A recently published RNA launch system enables the study of HBV replication and could help identify

consecutive off-treatment time points or measured at the last single off-treatment time point. (D) Mean (SE) change from EOT in HBsAg levels by off-treatment VR and presence or absence of either S- or X-trigger target region substitutions. BL, baseline; EOT, end of treatment; ETV, entecavir; HBeAg, hepatitis B e antigen; HBsAg, hepatitis B surface antigen; HBV DNA, hepatitis B DNA; NA, nucleos(t)ide analogue; NCT, not currently treated; ND, not determinable; PBO, placebo; POI, positions of interest (nt 261-278 for S-trigger and nt1781-1798 for X-trigger); SCR, Screening; SE, standard error; Subst, nt-substitution; TDF/TAF, tenofovir; TW, treatment week; VR, viral relapse; VS, virologically suppressed; w/, with; w/o, without.

sequence variants that confer resistance to antiviral drugs, including antisense oligonucleotides.[25]

Importantly, the detection of S-/X-trigger substitutions in JNJ-3989-treated patients was not associated with differences in off-treatment HBsAg kinetics. In addition, there was no difference in the time to VR between JNJ-3989-treated patients with and without observed S-/X-trigger variants. Importantly, HBV DNA increases were less frequent and less pronounced in REEF-2 active arm patients *vs.* NA-control arm patients, resulting in a lower rate of ALT flares.[2] Notably, the detection of S-/X-trigger target variants did not lead to more patients experiencing VR with higher peak HBV DNA values, requiring NA treatment to restart.

In conclusion, baseline nucleotide polymorphisms in the JNJ-3989 S-/X-trigger target regions, relevant for siRNA-induced RNA silencing, were found in 10% and 2.4% of NCT patients, respectively. These polymorphisms did not explain the variability in HBsAg and HBeAg declines during JNJ-3989 treatment. In patients who discontinued treatment and experienced VR, X-trigger substitutions were observed post-VR, likely arising from increased HBV replication after treatment cessation, without affecting off-treatment HBsAg kinetics. JNJ-3989 (DAP/TOM) is under investigation alongside the antisense oligonucleotide bepirovirsen in the B-United study (NCT06537414). The effects of X-trigger substitutions on re-treatment with JNJ-3989 or other RNA-based therapies remain unknown.

Affiliations

[1]*Johnson & Johnson, Beerse, Belgium;* [2]*Johnson & Johnson, Titusville, United States;* [3]*Johnson & Johnson, High Wycombe, United Kingdom;* [4]*Institute of Liver Studies, King's College Hospital, London, United Kingdom;* [5]*Department of Medicine, The University of Hong Kong, Hong Kong, China*

Abbreviations

ALT, alanine aminotransferase; CHB, chronic hepatitis B; HBeAg, hepatitis B e antigen; HBsAg, hepatitis B surface antigen; JNJ-3989, JNJ-73763989; JNJ-6379, JNJ-56136379; NA, nucleos(t)ide analogue; NCT, not currently treated; nt, nucleotide; POI, position of interest; siRNA, small-interfering RNA; VR, viral relapse; VS, virologically suppressed.

Financial support

This study was sponsored by Johnson & Johnson with medical writing support provided by Lumanity Communications Inc., which was funded by Johnson & Johnson.

Conflicts of interest

TV, EL, JJ, MB, IA, CG, SDM, and OL are or were employees of Johnson & Johnson and may hold stock in Johnson & Johnson. OL is current employee of GSK and may hold stock in GSK. KA received grants from Abbott and MSD; served as a consultant for Johnson & Johnson, Assembly, Arbutus, Immunocore, Roche, BMS, Boehringer Ingelheim, Novartis, Shinoigi, and Sobi; served as a speaker for Gilead and Sobi; and served on a data safety monitoring or advisory board for Drug Farm, NUC-B, and Aligos. M-FY served as advisor/consultant for AbbVie, AlloVir International, Arbutus Biopharma, Bristol Myers Squibb, ClearB Therapeutics, Dicerna Pharmaceuticals, Gilead Sciences, GlaxoSmithKline, Johnson & Johnson, Merck Sharp & Dohme, Roche, and Spring Bank Pharmaceuticals; and received grant/research support from Assembly Biosciences, Arrowhead Pharmaceuticals, Bristol Myers Squibb, Fujirebio Diagnostics Inc., Gilead Sciences, Merck Sharp & Dohme, Roche, Spring Bank Pharmaceuticals, and Sysmex Corp.

Please refer to the accompanying ICMJE disclosure forms for further details.

Authors' contributions

All authors were involved in the critical revisions of the manuscript and the review of important content, were accountable for all aspects of the work (accuracy and integrity), and approved the final submitted manuscript. At least one author had access to all of the data and can vouch for the integrity of the data analyses. TV, EL, JJ, MB, IA, CG, SDM, and OL designed the protocol and analyzed the data.

Data availability

The data sharing policy of Janssen Pharmaceutical Companies of Johnson & Johnson is available at https://www.janssen.com/clinical-trials/transparency. As noted on this site, requests for access to the study data can be submitted through Yale Open Data Access (YODA) Project site at http://yoda.yale.edu.

Acknowledgments

The authors thank the patients in these studies and the other Johnson & Johnson staff members for their contributions to the studies. We thank Thomas Kakuda for his expert input and careful review of this manuscript. This study was sponsored by Johnson & Johnson, with editorial support provided by Kim Caldwell, PhD, of Lumanity Communications Inc., and was funded by Johnson & Johnson.

Supplementary data

Supplementary data to this article can be found online at https://doi.org/10.1016/j.jhepr.2025.101618.

References

Author names in bold designate shared co-first authorship

[1] Wooddell CI, Lenz O, Schluep T, et al. Discovery and development of ARO-HBV/JNJ-3989. Trends Antivir Drug Develop 2025:211–246. Edition 1, Chapter 6.

[2] Agarwal K, Buti M, van Bommel F, et al. JNJ-73763989 and bersacapavir treatment in nucleos(t)ide analogue-suppressed patients with chronic hepatitis B: REEF-2. J Hepatol 2024;81(3):404–414.

[3] Yuen MF, Asselah T, Jacobson IM, et al. Efficacy and safety of the siRNA JNJ-73763989 and the capsid assembly modulator JNJ-56136379 (bersacapavir) with nucleos(t)ide analogues for the treatment of chronic hepatitis B virus infection (REEF-1): a multicentre, double-blind, active-controlled, randomised, phase 2b trial. Lancet Gastroenterol Hepatol 2023;8(9):790–802.

[4] Birmingham A, Anderson E, Sullivan K, et al. A protocol for designing siRNAs with high functionality and specificity. Nat Protoc 2007;2(9):2068–2078.

[5] Jackson AL, Linsley PS. Recognizing and avoiding siRNA off-target effects for target identification and therapeutic application. Nat Rev Drug Discov 2010;9(1):57–67.

[6] Wee LM, Flores-Jasso CF, Salomon WE, et al. Argonaute divides its RNA guide into domains with distinct functions and RNA-binding properties. Cell 2012;151(5):1055–1067.

[7] **Sheu-Gruttadauria J**, **Xiao Y**, Gebert LF, et al. Beyond the seed: structural basis for supplementary microRNA targeting by human Argonaute2. EMBO J 2019;38(13):e101153.

[8] Sarrazin C. Treatment failure with DAA therapy: importance of resistance. J Hepatol 2021;74(6):1472–1482.

[9] Carr A, Mackie NE, Paredes R, et al. HIV drug resistance in the era of contemporary antiretroviral therapy: a clinical perspective. Antivir Ther 2023;28(5):13596535231201162.

[10] Zoulim F, Locarnini S. Hepatitis B virus resistance to nucleos(t)ide analogues. Gastroenterology 2009;137(5). 1593-608 e1-2.

[11] Boden D, Pusch O, Lee F, et al. Human immunodeficiency virus type 1 escape from RNA interference. J Virol 2003;77(21):11531–11535.

[12] Wilson JA, Richardson CD. Hepatitis C virus replicons escape RNA interference induced by a short interfering RNA directed against the NS5b coding region. J Virol 2005;79(11):7050–7058.

[13] Gitlin L, Stone JK, Andino R. Poliovirus escape from RNA interference: short interfering RNA-target recognition and implications for therapeutic approaches. J Virol 2005;79(2):1027–1035.

[14] FDA. Submitting next generation sequencing data to the division of antiviral products. In: US Department of Health and Human Services FaDAF, Center for Drug Evaluation and Research (CDER), editor. https://www.fda.gov/regulatory-information/search-fda-guidance-documents/submitting-next-generation-sequencing-data-division-antiviral-products-guidance-industry-technical2019.
[15] **Schwarz DS**, **Ding H**, Kennington L, et al. Designing siRNA that distinguish between genes that differ by a single nucleotide. Plos Genet 2006;2(9):e140.
[16] Huang H, Qiao R, Zhao D, et al. Profiling of mismatch discrimination in RNAi enabled rational design of allele-specific siRNAs. Nucleic Acids Res 2009;37(22):7560–7569.
[17] **van Bommel F**, **Bartens A**, Mysickova A, et al. Serum hepatitis B virus RNA levels as an early predictor of hepatitis B envelope antigen seroconversion during treatment with polymerase inhibitors. Hepatology 2015;61(1):66–76.
[18] Mak LY, Wooddell CI, Lenz O, et al. Long-term hepatitis B surface antigen response after finite treatment of ARC-520 or JNJ-3989. Gut 2025;74(3):440–450.
[19] Seeger C, Mason WS. Molecular biology of hepatitis B virus infection. Virology 2015;479–480:672–686.
[20] Asselah T, Kakuda TN, Vilgrain V, et al. Liver concentrations of JNJ-73763989 in patients with chronic hepatitis B: the INSIGHT Study Panel 3 results. Los Cabos: HepDART; 2023.
[21] Sandra L, T'Jollyn H, Goeyvaerts N, et al. Plasma and liver pharmacokinetics of the N-acetylgalactosamine short interfering RNA JNJ-73763989 in recombinant adeno-associated-hepatitis B virus-infected Mice. J Pharmacol Exp Ther 2022;383(1):70–79.
[22] Yan JH, Bifano M, Olsen S, et al. Entecavir pharmacokinetics, safety, and tolerability after multiple ascending doses in healthy subjects. J Clin Pharmacol 2006;46(11):1250–1258.
[23] Kearney BP, Flaherty JF, Shah J. Tenofovir disoproxil fumarate: clinical pharmacology and pharmacokinetics. Clin Pharmacokinet 2004;43(9):595–612.
[24] Zoulim F, Lenz O, Vandenbossche JJ, et al. JNJ-56136379, an HBV capsid assembly modulator, is well-tolerated and has antiviral activity in a phase 1 study of patients with chronic infection. Gastroenterology 2020;159(2):521–533 e9.
[25] **Yu Y**, **Schneider WM**, Kass MA, et al. An RNA-based system to study hepatitis B virus replication and evaluate antivirals. Sci Adv 2023;9(15):eadg6265.

Keywords: hepatitis B; siRNA; genome sequencing; JNJ-73763989; DAP/TOM; polymorphisms; viral breakthrough; viral relapse; HBsAg.

Received 3 April 2025; received in revised form 22 September 2025; accepted 29 September 2025; Available online 9 October 2025

Journal of Hepatology, Volume *7*

Supplemental information

Viral sequence analysis of chronic hepatitis B patients treated with the siRNA JNJ-73763989 in phase II clinical trials

Thierry Verbinnen, Erkki Lathouwers, John Jezorwski, Michael Biermer, Ilse Augustyns, Craig Grant, Kosh Agarwal, Man-Fung Yuen, Sandra De Meyer, and Oliver Lenz

Viral sequence analysis of chronic hepatitis B patients treated with the siRNA JNJ-73763989 in phase II clinical trials

Thierry Verbinnen, Erkki Lathouwers, John Jezorwski, Michael Biermer, Ilse Augustyns, Craig Grant, Kosh Agarwal, Man-Fung Yuen, Sandra De Meyer, Oliver Lenz

Table of contents

Supplementary methods……………………………………………………………2

Supplementary tables………………………………………………………….…...5

Supplementary figures……………………………………………………………14

Supplementary references………………………………………………………..19

Supplementary methods

HBV Genome Sequencing and Genotyping

HBV genome sequencing was performed at DDL Diagnostic Laboratory/Cerba Research (the Netherlands). Total nucleic acids were isolated from 200 µL of plasma and eluted in 20-50 µL of eluate. The isolated DNA was amplified by PCR and nested PCR, using the Expand high-fidelity PCR kit (Roche Molecular Systems). 10 µL of DNA was used for outer PCR. Primers for HBV full genome amplification are in the preCore and the X-gene. If the initial amplification for the full genome large fragment failed, an alternative approach, amplifying the HBV full genome in 2 smaller fragments (HBV full genome partial 1/2) was tested. PCR products were analyzed on a Qiaxel capillary system using standard protocols, to confirm successful amplification of a PCR fragment of the expected size, and to determine whether the quantity of product is sufficient for successful sequence analysis. The PCR products that showed a clear band of the expected size after gel-electrophoresis were cleaned up by Ampure XP beads (Beckman Coulter) to remove primer-dimers and small aspecific PCR products. The purified PCR products were quantified using the Quant-iT PicoGreen dsDNA kit (Life Technologies). The diluted PCR products were fragmented and tagged using the 'tagmentation' method (Nextera XT sample preparation kit). Index primers (Nextera XT Index kit) were added by limited cycle PCR. Prior to sample pooling, samples were normalized using beads with maximum binding capacity (Nextera XT sample preparation kit). The different PCR products were not pooled but sequenced separately. Sequence data from multiple overlapping fragments were subjected to primer trimming and subsequent in-silico pooling before further analysis. Sequencing was performed on the Illumina Miseq platform using the Miseq v2 sequencing kit with 300 cycles (Illumina). De-multiplexed FASTQ files were generated as an output.

Apart from baseline samples, which were sequenced for all patients with sufficiently high HBV DNA levels, sequencing of post-baseline samples was initiated based on changes in

HBV DNA levels observed in each patient and the limits of the sequencing assay. If both the main and the back-up HBV full genome sequence approaches described above failed, exploratory sub-genomic sequencing approaches were attempted. These consisted of either the separate sequencing of partial 1 or partial 2 fragments, the precore/core region, and/or an exploratory short fragment 3 (SF3) (Figure S3).

Baseline polymorphisms included amino acid and nucleotide changes from the universal HBV genotype-A reference sequence (National Center for Biotechnology Information ID X02763) at baseline with a sequence-read frequency ≥15%. Emerging amino acid and nucleotide substitutions included substitutions detected post-baseline with a sequence-read frequency ≥15%, while not present at baseline (read frequency <1%). The sequence-read frequency cutoffs used were consistent with those proposed by the Food and Drug Administration (1).

HBV RNA-based Sequencing and HBV Genotyping

HBV RNA-based sequencing and genotyping was performed at DDL Diagnostic Laboratory/Cerba Research (the Netherlands). Genotyping of HBV-RNA was performed by analysis of the complete RT-domain, which contains sufficient sequence variation to allow identification of all specific HBV GT (Figure S4). After total nucleic acid extraction from the serum or plasma sample, reverse transcription of the RNA was performed by using an HBV-RNA specific RT-primer. Amplification of the RT-domain was performed in two consecutive amplification rounds. Positive RT-domain amplimers were sequenced by ultra-deep sequencing (UDS by Illumina MiSeq), and the HBV GT was identified by phylogenetic analysis comparing the (25% consensus) sequence to HBV GT reference sequences.

The analytical and clinical sensitivity of the assay was tested on dilution series of HBV in vitro transcribed (IVT) RNA, synthesized from HBV plasmid DNA (GT-D), and of 5 plasma samples with known historical HBV GT-A/B/C/D/F. IVT RNA and plasma samples could be amplified

and genotyped when HBV RNA levels were ≥ 2.5 log10 cps/mL. HBV RNA-based GT could be established in 23 of 26 clinical samples (Figure S4) with HBV-RNA concentrations ranging from 2.4 – 6.0 log10 cps/mL (3 failed samples had HBV RNA < 3.5 log10 cps/mL). Majority of samples tested (n=21) had HBV-DNA concentrations < 65 IU/mL, 5 samples had > 20,000 IU/mL.

Comparison of the HBV-RNA-based and prior determined HBV DNA-based GT result showed perfect match. The percentage identity between the HBV-DNA and RNA derived RT-domain sequences ranged between 99.3% and 100%.

Supplementary tables

Table S1: REEF-1 Patients with Baseline Nucleotide Polymorphisms in the JNJ-3989 S- and/or X-Trigger Target Region POIs 2-18 - by Treatment History and HBeAg Status

	NCT / HBeAg-positive	NCT / HBeAg-negative	Total NCT	VS / HBeAg-positive	VS / HBeAg-negative	Total VS
ITT	75	97	172	67	231	298
Patients with S-gene sequencing data, n	75†	93†	168†	26*	10*	36*
No baseline polymorphism, n (%)	71 (94.7)	80 (86.0)	151 (89.9)	26	5 (50.0)	31 (86.1)
1 or more baseline polymorphism, n (%)	4 (5.3)	13 (14.0)	17 (10.1)	0	5 (50.0)	5 (13.9)
273						
A273A/G	3 (4.0)	9 (9.7)	12 (7.1)	-	1 (10.0)	1 (2.8)
A273G	-	4 (4.3)	4 (2.4)	-	3 (30.0)	3 (8.3)
273+276				-		
A273A/G+T276T/C	1 (1.3)	-	1 (0.6)	-	-	-
A273G+T276T/C	-	-	-	-	1 (10.0)	1 (2.8)
Patients with X-gene sequencing data, n	75†	90†	165	28*	13*	41
No baseline polymorphism, n (%)	73 (97.3)	88 (97.8)	161 (97.6)	28 (100)	13 (100)	41 (100)
1 or more baseline polymorphism, n (%)	2 (2.7)	2 (2.2)	4 (2.4)	0 (0.0)	0 (0.0)	0 (0.0)
1794 (T1794T/A)	-	2 (2.2)	2 (1.2)	-	-	-
1795 (T1795T/A)	2 (2.7)	-	2 (1.2)	-	-	-

HBeAg, hepatitis B e antigen. ITT, intent-to-treat. NCT, not currently treated. POI, position of interest. VS, virologically suppressed.

The reference sequence used was the HBV genotype A universal reference sequence (NCBI ID X02763). Baseline polymorphisms were defined as changes from the universal HBV genotype A (NCBI ID X02763) reference sequence and if sequence read frequency was >15%.

The S- and X-trigger target region POI within the S- and X-genes were nt261-278 and nt1781-1798 (HBV full genome numbering based on EcoRI restriction site), respectively.

†HBV DNA-based sequencing was applied to baseline samples of NCT patients (ie, with HBV DNA >2,000 [HBeAg-negative] and >20,000 IU/mL [HBeAg-positive] at screening).

*HBV RNA based sequencing was applied to subset of baseline samples of VS patients (ie, patients with HBV DNA <60 IU/mL at screening and baseline HBV RNA ≥3.0 log_{10} copies/mL).

Table S2: Proportion of VS Patients with VR and Nucleotide Substitutions Observed at ≥1 S-trigger Target Region POI at Time of VR and During Aggregated Off-treatment Follow-up Period - by Study and Treatment

	REEF-1 JNJ-3989		REEF-1 JNJ-3989+JNJ-6379		REEF-2 JNJ-3989+JNJ-6379		Total JNJ-3989 (±JNJ-6379)		REEF-2 NA-control arm	
VS patients, mITT, n	176		61		84		321		44	
Patients who stopped all treatment (including NA), n	39		11		77		127		40	
Patients with VR, n (%)	17 (43.6)		5 (45.5)		53 (68.8)		75 (59.1)		34 (85)	
Patients with HBV DNA >200 IU/mL at single off-treatment time point, n (%)	4 (10.3)		4 (36.4)		1 (1.3)		9 (7.1)		5 (12.5)	
Time point of viral sequencing	VR+12w[†]	Aggregated FU[‡]	VR+12w[†]	Aggregated FU[‡]	VR+12w[†]	Aggregated FU[‡]	VR+12w[†]	Aggregated FU[‡]	VR+12w[†]	Aggregated FU[‡]
Patients with S-gene sequence info available, n	17	21	5	8	48	49	70	78	33	36

No substitutions at S-trigger POI 2-18, n (%)	14 (82.4)	13 (61.9)	5 (100)	7 (87.5)	34 (70.8)	33 (67.3)	53 (75.7)	53 (67.9)	28 (84.8)	29 (80.6)
≥1 substitutions at S-trigger POI 2-18, n (%)*	3 (17.6)	8 (38.1)	0 (0.0)	1 (12.5)	14 (29.2)	16 (32.7)	17 (24.3)	25 (32.1)	5 (15.2)	7 (19.4)
264	-	-	-	-	1 (2.1)	1 (2.0)	1 (1.4)	1 (1.3)	-	-
C264C/T	-	-	-	-	1 (2.1)		1 (1.4)	-	-	-
273	2 (11.8)	6 (28.6)	-	1 (12.5)	14 (29.2)	15 (30.6)	16 (22.9)	22 (28.2)	5 (15.2)	7 (19.4)
A273A/G	-	1 (4.8)	-	-	5 (10.4)	6 (12.2)	5 (7.1)	7 (9.0)	1 (3.0)	2 (5.6)
A273G	2 (11.8)	5 (23.8)	-	1 (12.5)	9 (18.8)	13 (26.5)	11 (15.7)	19 (24.4)	4 (12.1)	5 (13.9)
276	1 (5.9)	2 (9.5)	-	-	-	1 (2.0)	1 (1.4)	3 (3.8)	-	1 (2.8)
T276T/C	-	1 (4.8)	-	-	-	1 (2.0)	-	2 (2.6)	-	1 (2.8)
T276C	1 (5.9)	1 (4.8)	-	-	-	-	1 (1.4)	1 (1.3)	-	-

mITT, modified intent-to-treat. NA, nucleos(t)ide analogue. nt, nucleotide. POI, position of interest. VR, viral relapse (ie confirmed off-treatment increase in HBV DNA >200 IU/mL in patients with HBV DNA <LLOQ at EOT). VS, virologically suppressed. FU, follow-up.

Observed variants are defined as changes from the universal HBV genotype A (NCBI ID X02763) reference sequence and if sequence read frequency was >15%. Viral sequencing data included in the analysis was considering either 1) the first assessment up to and including 12-weeks after viral flare was identified (†VR+12w), or 2) all off-treatment time points with sequencing information available (‡Aggregated FU).

*A single patient could have 1 or more nucleotide substitutions observed at 1 or more S- or X-trigger POI during aggregated off-treatment follow-up period.

Table S3: Proportion of VS Patients with VR and Nucleotide Substitutions Observed at ≥1 X-trigger Target Region POI at Time of VR and During Aggregated Off-treatment Follow-up Period - by Study and Treatment

	REEF-1 JNJ-3989		REEF-1 JNJ-3989+JNJ-6379		REEF-2 JNJ-3989+JNJ-6379		Total JNJ-3989(±JNJ-6379)		REEF-2 NA-control arm	
VS patients, mITT	176		61		84		321		44	
Patients who stopped all treatment (including NA), n	39		11		77[†]		127		40[†]	
Patients with VR, n (%)	17 (43.6)		5 (45.5)		53 (68.8)		75 (59.1)		34 (85)	
Patients with HBV DNA >200 IU/mL at single off-treatment time point, n (%)	4 (10.3)		4 (36.4)		1 (1.3)		9 (7.1)		5 (12.5)	
Time point of viral sequencing	VR+ 12w[†]	Aggregated FU[‡]	VR+ 12w[†]	Aggregated FU[‡]	VR+ 12w[†]	Aggregated FU[‡]	VR+ 12w[†]	Aggregated FU[‡]	VR+ 12w[†]	Aggregated FU[‡]
Patients with X-gene sequence info available, n	17	21	4	7	48	49	69	77	33	35
No substitutions at X-trigger POI 2-18, n (%)	13 (76.5)	12 (57.1)	4 (100)	4 (57.1)	31 (64.6)	18 (36.7)	48 (69.6)	34 (44.2)	31 (94.0)	33 (94.3)
≥1 substitutions at X-trigger POI 2-18, n (%)*	4 (23.5)	9 (42.9)	0 (0.0)	3 (42.9)	17 (35.4)	31 (63.3)	21 (30.4)	43 (55.8)	2 (6.0)	2 (5.7)
1784	1 (5.9)	3 (14.3)	-	1 (14.3)	1 (2.1)	1 (2.0)	2 (2.9)	5 (6.5)	-	-
T1784T/C	1 (5.9)	1 (4.8)	-	-	-	1 (2.0)	1 (1.4)	3 (3.9)	-	-
T1784C		3 (14.3)	-	1 (14.3)	1 (2.1)	1 (2.0)	1 (1.4)	4 (5.2)	-	-
1785	2 (11.8)	3 (14.3)	-	-	2 (4.2)	11 (22.4)	4 (5.8)	14 (18.2)	-	-
A1785A/C	1 (5.9)	1 (4.8)	-	-	-	1 (2.0)	1 (1.4)	2 (2.6)	-	-

	REEF-1 JNJ-3989		REEF-1 JNJ-3989+JNJ-6379		REEF-2 JNJ-3989+JNJ-6379		Total JNJ-3989(±JNJ-6379)		REEF-2 NA-control arm	
A1785C	-	-	-	-	-	4 (8.2)	-	4 (5.2)	-	-
A1785A/G	-	-	-	-	1 (2.1)	5 (10.2)	1 (1.4)	5 (6.5)	-	-
A1785A/T	-	1 (4.8)	-	-	-	1 (2.0)	-	2 (2.6)	-	-
A1785C/T	-	-	-	-	-	1 (2.0)	-	1 (1.3)	-	-
A1785T	1 (5.9)	1 (4.8)	-	-	1 (2.1)	1 (2.0)	2 (2.9)	2 (2.6)	-	-
1787	-	-	-	-	-	1 (2.0)	-	1 (1.3)	-	-
G1787A	-	-	-	-	-	1 (2.0)	-	1 (1.3)	-	-
1793	1 (5.9)	1 (4.8)	-	-	-	-	1 (1.4)	1 (1.3)	-	-
A1793G	1 (5.9	1 (4.8)	-	-	-	-	1 (1.4)	1 (1.3)	-	-
1794	1 (5.9)	4 (19.0)	-	2 (28.6)	14 (29.2)	27 (55.1)	15 (21.7)	33 (42.9)	1 (3.0)	1 (2.9)
T1794T/A	1 (5.9)	3 (14.3)	-	-	6 (12.5)	17 (34.7)	7 (10.1)	20 (26.0)	-	-
T1794A	-	1 (4.8)	-	1 (14.3)	8 (16.7)	19 (38.8)	8 (11.6)	21 (27.3)	1 (3.0)	1 (2.9)
T1794T/G	-	1 (4.8)	-	1 (14.3)	1 (2.1)	3 (6.1)	1 (1.4)	5 (6.5)	-	-
T1794G	-	1 (4.8)	-	-	-	2 (4.1)	-	3 (3.9)	-	-
1795	-	1 (4.8)	-	-	-	3 (6.1)	-	4 (5.2)	-	-
T1795T/G	-	-	-	-	-	2 (4.1)	-	2 (2.6)	-	-
T1795G	-	1 (4.8)	-	-	-	1 (2.0)	-	2 (2.6)	-	-
1796	-	-	-	-	-	1 (2.0)	-	1 (1.3)	-	-
G1796G/A	-	-	-	-	-	1 (2.0)	-	1 (1.3)	-	-
1797	-	-	-	-	-	-	-	-	1 (3.0)	1 (2.9)
G1797G/A	-	-	-	-	-	-	-	-	1 (3.0)	1 (2.9)
G1797A	-	-	-	-	-	-	-	-	1 (3.0)	1 (2.9)

EOT, end of treatment. HBV, hepatitis B virus. mITT, modified intent-to-treat. NA, nucleos(t)ide analogue. nt, nucleotide. POI, position of interest. VR, viral relapse (ie, confirmed off-treatment increase in HBV DNA >200 IU/mL in patients with HBV DNA <LLOQ at EOT). VS, virologically suppressed. FU, follow-up.

	REEF-1 JNJ-3989	REEF-1 JNJ-3989+JNJ-6379	REEF-2 JNJ-3989+JNJ-6379	Total JNJ-3989(±JNJ-6379)	REEF-2 NA-control arm

Observed variants are defined as changes from the universal HBV genotype A (NCBI ID X02763) reference sequence and if sequence read frequency was >15%.

Viral sequencing data included in the analysis was considering either 1) the first assessment up to and including 12-weeks after viral flare was identified (†VR+12w), or 2) all off-treatment time points with sequencing information available (‡Aggregated FU).

*A single patient could have 1 or more nucleotide substitutions observed at 1 or more S- or X-trigger POI during aggregated off-treatment follow-up period.

†In REEF-2, 7/84 active arm and 4/44 control arm patients either did not stop NA or discontinued study treatment early (2).

Table S4: Median (Range) Time from End-of-Treatment to VR in VS HBeAg-negative patients (REEF-2) - by Treatment and Presence of S- (Top) and X- (Bottom) Trigger Target Region nt Substitutions

Median (range) Time to VR, days	ETV as backbone NA	TDF/TAF as backbone NA
PBO+NA	112 (54-196; N=14)	29 (15-56; N=20)
JNJ-3989+JNJ-6379+NA	144 (57-257; N=17)	153 (28-337; N=36)
with X substitution	140 (57-257; n=10)	141 (28-309; n=21)
without X substitution	169 (140-212; n=6)	139 (56-337; n=12)
with S substitution	126 (57-207; n=6)	167 (28-309; n=10)
without S substitution	156 (115-257; n=10)	113 (28-337; n=23)
No sequence info available*	224 (ND; n=1)	296 (295-337; n=3)

NA, nucleos(t)ide analogue. PBO, placebo. EOT, end of treatment. ETV, entecavir. VR, viral relapse. VS, virologically suppressed. TDF/TAF, tenofovir. ND, not determinable.

N, number of patients with VR (ie, confirmed off-treatment HBV DNA >200 IU/mL).

n, number of patients with VR and with off-treatment sequence data available. *Sequencing of off-treatment samples failed for 4 JNJ-3989-treated patients with VR.

Time to VR was calculated relative to end-of-treatment visit and was considered the first off-treatment time point with HBV DNA >200 IU/mL.

The S- and X-trigger target region positions of interest (POI) within the S- and X-genes were nt261-278 and nt1781-1798 (HBV full genome numbering based on EcoRI restriction site), respectively.

Table S5: Prevalence Among HBV Sequences in Public Database

Variant	Prevalence
S: A273G	771/17795 (4.3%)
X: T1794A	10/5947 (0.17%)
X: T1794G	4/5947 (0.07%)
X: T1795G	10/5947 (0.17%)
X: T1784C	7/5947 (0.12%)
X: A1785C	6/5947 (0.10%)
X: A1785G	0/5947 (0.0%)
X: A1785T	13/5947 (0.22%)

HBV, hepatitis B virus.

HBVdb public HBV sequence database can be accessed via https://hbvdb.ibcp.fr/HBVdb/ (HBVdb export of sequences included in this report was performed in April, 2016) (3)

Supplementary figures

Fig. S1: REEF-1 study design (4).

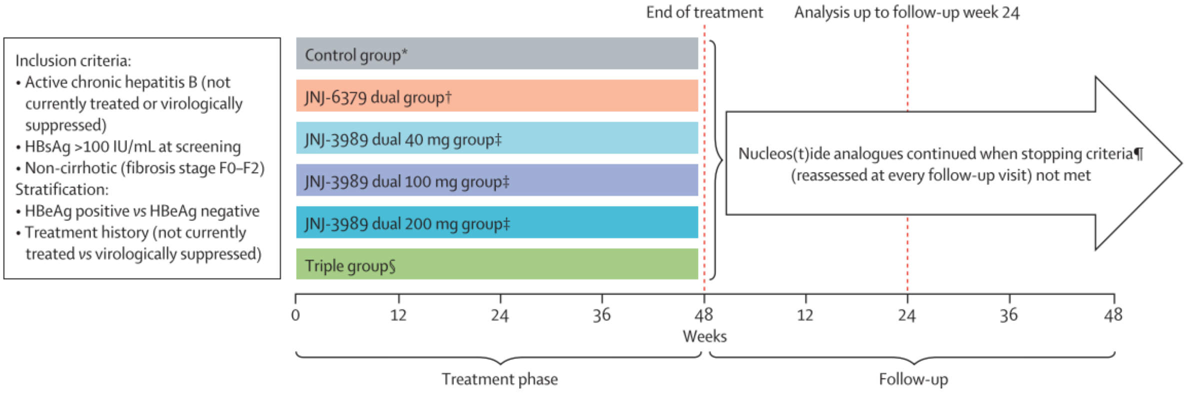

HBeAg, hepatitis B e antigen.

Fig. S2: REEF-2 study design (2).

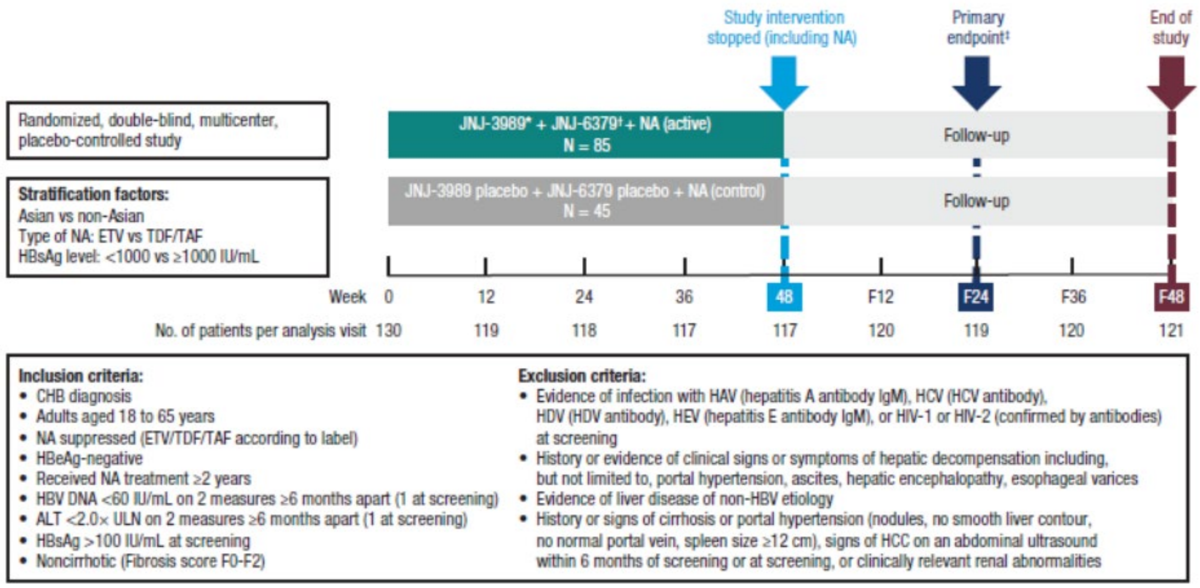

ALT, alanine transaminase; CHB, chronic hepatitis B; ETV, entecavir; F, follow-up; HAV, hepatitis A virus; HBeAg, hepatitis B e antigen; HBsAg, hepatitis B surface antigen; HCC, hepatocellular carcinoma; HCV, hepatitis C virus; HDV, hepatitis D virus, HEV, hepatitis E virus; HIV, human immunodeficiency virus; JNJ-3989, JNJ-73763989; JNJ-6379, JNJ-56136379; LLOQ, lower limit of quantification; NA, nucleos(t)ide analog; PO, oral; SC, subcutaneous; TAF, tenofovir alafenamide; TDF, tenofovir disoproxil fumarate; ULN, upper limit of normal.

*200 mg SC every 4 weeks.

†250 mg PO daily.

‡HBsAg seroclearance (HBsAg <LLOQ [0.05 IU/mL]) at Week 72 without restarting NA treatment.

Fig. S3: HBV genomic organization and sequencing strategies.

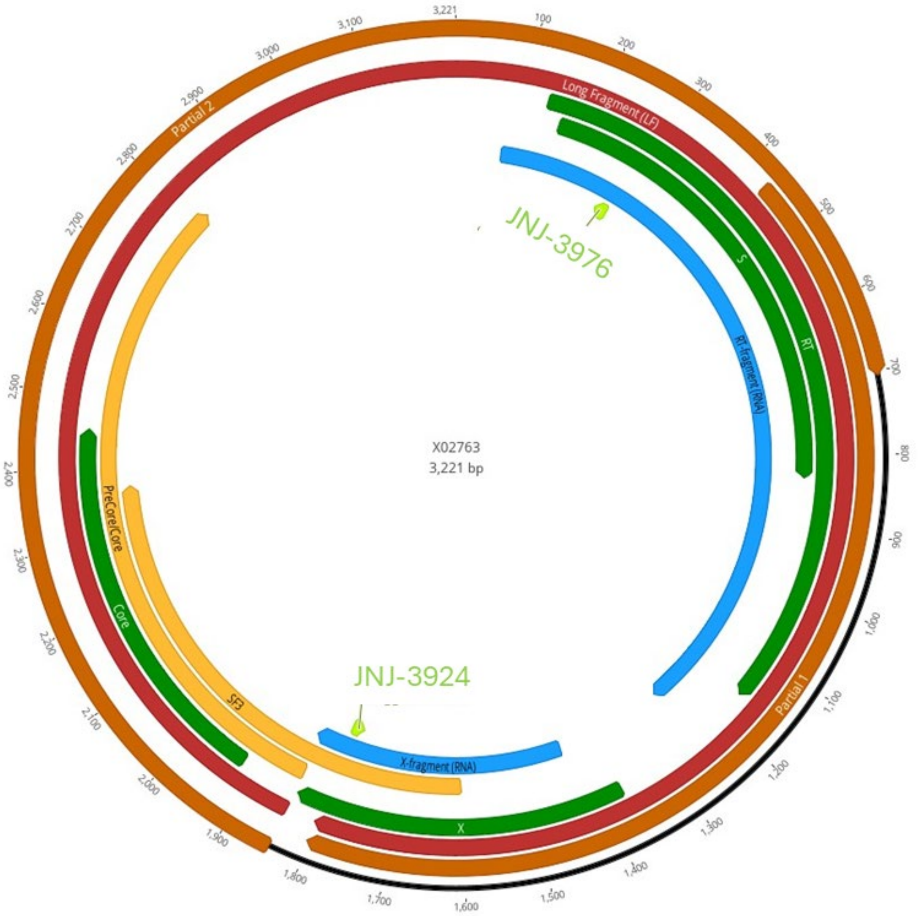

HBV genome sequence shown is the universal HBV genotype A reference sequence (NCBI ID X02763). HBV genetic regions of interest HBV core, RT-domain, S- and X-gene are shown in green. Nucleotide numbering based on EcoR1 restriction site.

Main and back-up HBV full genome sequencing approaches are shown in red (long fragment [LF]) and orange (BU LF with partial 1 and partial 2 overlapping fragments). Exploratory HBV DNA based sub-genomic sequencing approaches are shown in yellow (i.e., PreCore/Core and Short fragment 3 [SF3)]. Exploratory HBV RNA based sub-genomic sequence approaches are shown in blue (i.e., RT- and X-fragment).

JNJ-3976 ("S-trigger") and JNJ-3924 ("X-trigger") trigger target regions (nt261-268 and nt1781-1798, respectively) are indicated in light green highlight.

Alignment was generated using Geneious Prime.

Fig. S4: (a) Schematic representation of HBV RNA RT-qPCR and HBV RNA RT-domain amplification strategies and (b) Phylogenetic tree based on the 25% consensus sequences of the obtained (n = 23) HBV RNA genotypes (including the reference sequences from genotype A-I).

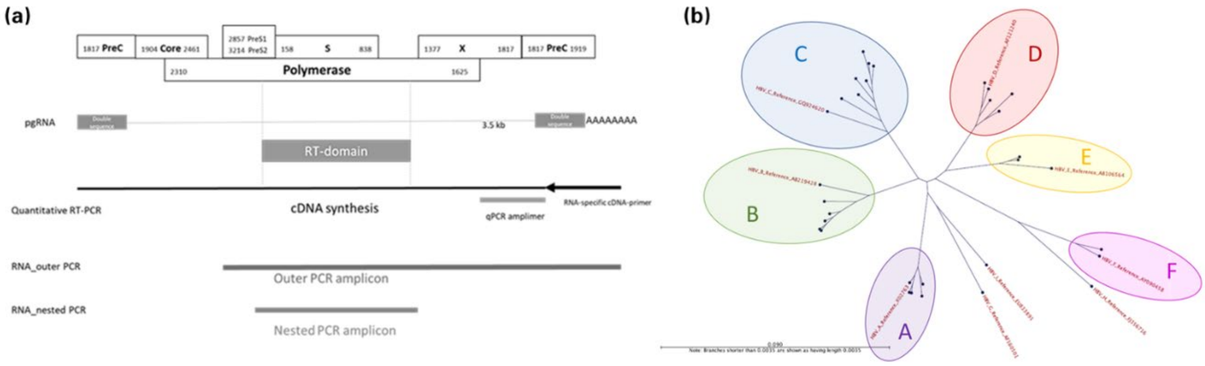

Fig. S5: Representative examples of VS, JNJ-3989-treated patients (all REEF-2) with VR and X-trigger region nt substitution(s) observed during off-treatment follow-up.

Patient 1

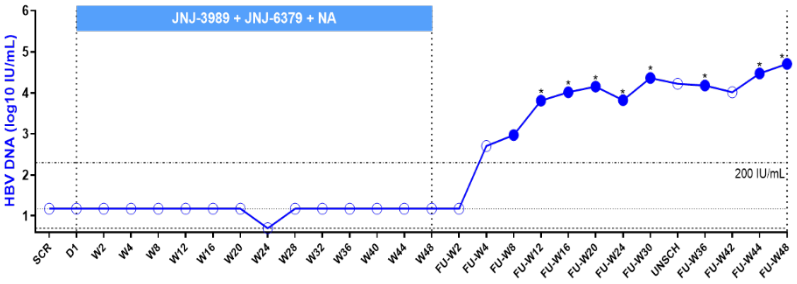

Substitution, %*	FU W8	FU W12	FU W16	FU W20	FU W24	FU W30	FU W36	FU W44	FU W48
**T1794A**	-	70.02	79.71	78.52	99.91	76.22	99.86	99.80	88.60

Patient 2

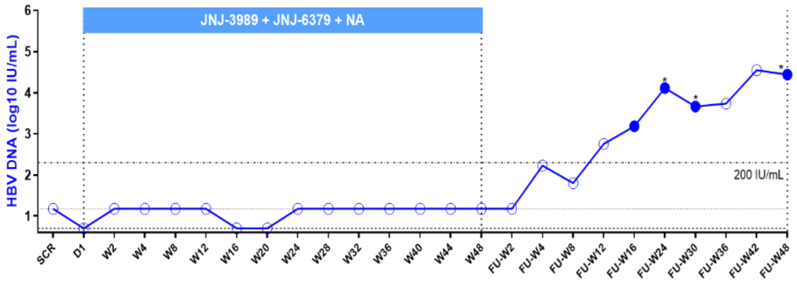

Substitution, %*	FU W16	FU W24	FU W30	FU W48
**T1794A**	6.89	51.98	85.76	80.99

Patient 3

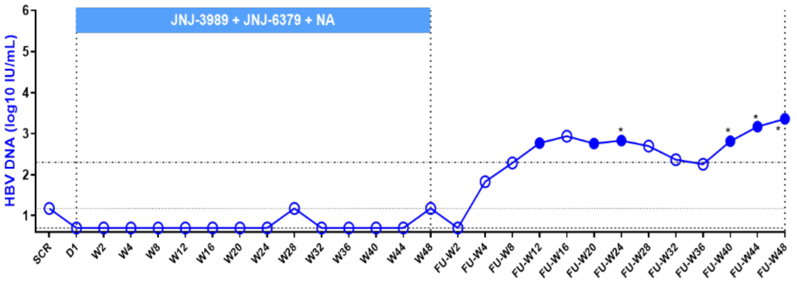

Substitution, %*	FU W12	FU W20	FU W24	FU W40	FU W44	FU W48
**T1794A**	4.74	6.84	99.86	99.86	99.79	99.86

Patient 4

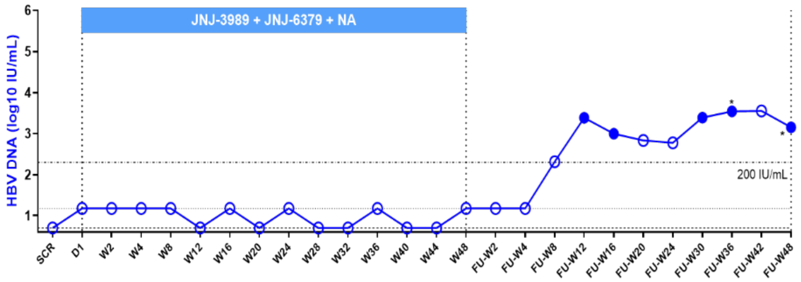

Substitution, %*	FU W12	FU W16	FU W30	FU W36	FU W48
**T1794A**	-	-	-	41.56	73.68

SCR, screening. D, day. FU-W, follow-up Week. NA, nucleos(t)ide analogue. “-”, sequenced but no variant observed.

Closed and open dots indicate visits with and without HBV sequence information available, respectively. Asterix (*) represents visit at which the nt substitution was observed with sequence-read frequency >15%.

*X-trigger region nt substitution of interest and Illumina MiSeq sequencing sequence read-frequencies (%) are shown. Wild type nt variant is not shown.

Supplementary references

1. FDA. Submitting next generation sequencing data to the division of antiviral products. In: US Depertment of Health and Human Services FaDAF, Center for Drug Evaluation and Research (CDER), editor. https://www.fda.gov/regulatory-information/search-fda-guidance-documents/submitting-next-generation-sequencing-data-division-antiviral-products-guidance-industry-technical2019.
2. Agarwal K, Buti M, van Bommel F, et al. JNJ-73763989 and bersacapavir treatment in nucleos(t)ide analogue-suppressed patients with chronic hepatitis B: REEF-2. J Hepatol. 2024;81(3):404-14.
3. Hayer J, Jadeau F, et al. HBVdb: a knowledge database for Hepatitis B Virus. Nucleic Acids Res. 2013 Jan;41.
4. Yuen MF, Asselah T, Jacobson IM, et al. Efficacy and safety of the siRNA JNJ-73763989 and the capsid assembly modulator JNJ-56136379 (bersacapavir) with nucleos(t)ide analogues for the treatment of chronic hepatitis B virus infection (REEF-1): a multicentre, double-blind, active-controlled, randomised, phase 2b trial. Lancet Gastroenterol Hepatol. 2023;8(9):790-802.
